# The cellular and molecular landscape of hypothalamic patterning and differentiation from embryonic to late postnatal development

**DOI:** 10.1038/s41467-020-18231-z

**Published:** 2020-08-31

**Authors:** Dong Won Kim, Parris Whitney Washington, Zoe Qianyi Wang, Sonia Hao Lin, Changyu Sun, Basma Taleb Ismail, Hong Wang, Lizhi Jiang, Seth Blackshaw

**Affiliations:** 1 Solomon H. Snyder Department of Neuroscience, Johns Hopkins University School of Medicine, Baltimore, MD 21205 USA; 2 Department of Ophthalmology, Johns Hopkins University School of Medicine, Baltimore, MD 21205 USA; 3 Department of Neurology, Johns Hopkins University School of Medicine, Baltimore, MD 21205 USA; 4 Center for Human Systems Biology, Johns Hopkins University School of Medicine, Baltimore, MD 21205 USA; 5 Institute for Cell Engineering, Johns Hopkins University School of Medicine, Baltimore, MD 21205 USA; 6 Kavli Neuroscience Discovery Institute, Johns Hopkins University School of Medicine, Baltimore, MD 21205 USA

**Keywords:** Cell fate and cell lineage, Neural patterning, Neural progenitors

## Abstract

The hypothalamus is a central regulator of many innate behaviors essential for survival, but the molecular mechanisms controlling hypothalamic patterning and cell fate specification are poorly understood. To identify genes that control hypothalamic development, we have used single-cell RNA sequencing (scRNA-Seq) to profile mouse hypothalamic gene expression across 12 developmental time points between embryonic day 10 and postnatal day 45. This identified genes that delineated clear developmental trajectories for all major hypothalamic cell types, and readily distinguished major regional subdivisions of the developing hypothalamus. By using our developmental dataset, we were able to rapidly annotate previously unidentified clusters from existing scRNA-Seq datasets collected during development and to identify the developmental origins of major neuronal populations of the ventromedial hypothalamus. We further show that our approach can rapidly and comprehensively characterize mutants that have altered hypothalamic patterning, identifying *Nkx2.1* as a negative regulator of prethalamic identity. These data serve as a resource for further studies of hypothalamic development, physiology, and dysfunction.

## Abstract (web summary)

The cellular and molecular mechanisms regulating hypothalamic patterning and differentiation are unclear. Here, the authors profiled the transcriptome of the developing hypothalamus at single cell level, providing a resource to hypothalamic development in health and disease.

## Introduction

The hypothalamus is comprised of a diverse array of neuronal and glial cell types, many of which are organized into spatially discrete clusters or nuclei^1–3^. Stereotactic lesion and focal stimulation studies have identified individual nuclei as essential for regulating a broad range of homeostatic physiological processes, ranging from circadian rhythms to hunger; behaviors such as mating, aggression and care of young; and cognitive processes such as motivation, reward, and memory^4–7^. More recently, opto- and chemogenetic techniques have made it possible to identify the role of individual hypothalamic neuronal subtypes in controlling some of these behaviors^8–10^.

Progress in this area has been hampered, however, by the fact that hypothalamic cell types thus far have remained quite poorly characterized, despite recent efforts aimed at using scRNA-seq to classify cells in different regions of the adult hypothalamus^11–15^, and more recently in limited embryonic and early postnatal periods^16,17^. Still less is known about how hypothalamic cell types acquire their identities during development. Even the basic spatial organization of the developing hypothalamus, and its relationship to other forebrain structures such as the prethalamus and telencephalon, remains contentious^18–20^. Previous efforts using microarray analysis coupled with large-scale two-color in situ hybridization (ISH) have identified a set of molecular markers that uniquely define spatial domains of the early embryonic hypothalamus and adjacent diencephalic regions^2^, while parallel efforts using high-throughput ISH have identified additional region-specific markers^21,22^.

These datasets have been used as the basis for genetic studies that selectively disrupt the development of specific hypothalamic regions and/or cell types^23–27^, leading to the identification of novel functions for previously characterized hypothalamic regions or cell types^28,29^. However, these datasets have important limitations: they do not provide cellular resolution of gene expression data, and they do not efficiently measure the coexpression of multiple genes. In addition, despite the availability of many highly specific molecular markers, analysis of mutants that affect hypothalamic development is currently both slow and difficult, owing to the complexity of this structure.

Recent advances in single-cell RNA-seq technology (scRNA-seq)^30^ have made it possible to both analyze the development of complex organs at cellular resolution and to also rapidly and comprehensively characterize the molecular phenotype of developmental mutants^31^. In this study, we use scRNA-seq to profile changes in gene expression and cell composition across the full course of mouse hypothalamic development, with a particular focus on identifying genes that control glial differentiation and function. We next focus on identifying genes that control hypothalamic regionalization and neurogenesis in the early embryo, and integrate these findings to generate a *Hy*pothalamic *D*evelopmental *D*atabase (HyDD), which identifies selective markers of each region of the developing hypothalamus and prethalamus. We next use the HyDD to rapidly annotate cell types in previously published scRNA-seq datasets, and to infer the developmental history of specific subtypes of adult hypothalamic neurons. Finally, we demonstrate how the HyDD can be used to comprehensively analyze developmental mutants that generate complex phenotypes that would be difficult to characterize with traditional histology-based approaches, and in the process identify *Nkx2-1* as a negative regulator of prethalamic identity.

This study provides a reference atlas for future studies of hypothalamic development. It also identifies pathways by which gene regulatory networks that control cell identity can be targeted to analyze the functional role of individual hypothalamic neuronal subtypes.

## Results

### Comprehensive profiling of entire hypothalamus development

**Fig. 1:**
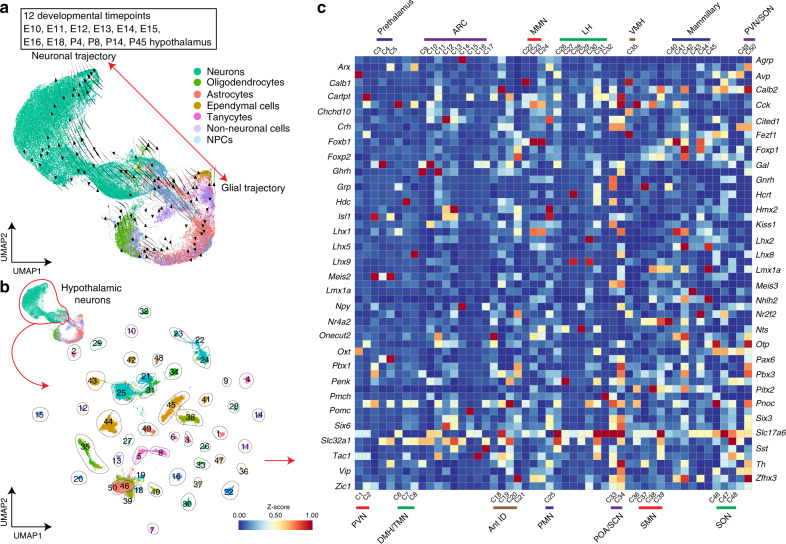
Overview of generation of the hypothalamus scRNA-seq dataset. **a** UMAP plot showing scRNA-seq data obtained from the developing diencephalon (including the prethalamus and hypothalamus) between E10 and E16, E18, P4, P8, P14, and P45. RNA velocity marks neuronal or glial (oligodendrocytes, astrocytes, ependymal cells, and tanycytes) trajectories. **b** UMAP plot showing neuronal clusters across the entire course of hypothalamus development. **c** Heatmap showing subtypes of neuronal clusters based on neuropeptide and transcription factor expression.

To profile changes in gene expression across the full course of mouse hypothalamic development, we processed 12 time points ranging from embryonic day (E)10 to postnatal day (P)45. For E10–E16, both prethalamus and hypothalamus were collected, whereas for E18–P45, only the hypothalamus was profiled (Fig. 1a, Supplementary Fig. 1). In total, 129,151 cells were profiled (Supplementary Fig. 2a, b). Using molecular markers of known hypothalamic regions and cell types^2^, we were able to annotate all major hypothalamic and adjacent brain regions, and major cell types at each individual age (Supplementary Fig. 2c, d, Supplementary Fig. 3a). Roughly similar detection of expressed genes and total mRNAs were observed at each time point (Supplementary Fig. 3b, c).

UMAP plotting paired with RNA velocity showed separate neuronal and glial trajectories (Fig. 1a), where glial trajectories in turn gave rise to trajectories leading to oligodendrocytes, astrocytes, ependymal cells, and tanycytes (Fig. 1a).

Trajectories leading from neuronal progenitors to mature neurons were then extracted to identify different subclusters of hypothalamic neurons (Fig. 1b). A total of 50 different major neuronal clusters across the hypothalamus were identified, distinguished by unique (*Agrp*, *Hcrt*, and *Pomc)* or shared (*Th*, *Gal*, and *Pnoc)* neuropeptides and neurotransmitter expression, as well as transcription factor expression that acts as a positional code to identify the location of these neuronal clusters (Fig. 1c). Expression of most neuropeptide and neurotransmitter-based markers is shared across multiple hypothalamic neuronal types, and most neuronal cluster identities were distinguished by their transcription factor-based positional codes (i.e., *Lhx9*+ and *Hcrt*+ LH neuronal cluster and *Lhx9*+ and *Pnoc*+ LH neuronal cluster). However, owing to the high cellular complexity of the hypothalamus, these neuronal clusters do not necessarily correspond to individual cell types, but rather to clusters of neurons that share common spatial location and/or developmental origin, which can be seen by the fact that several neuronal clusters show enriched expression for synaptic vesicle transporters for both glutamate (*Slc17a6*) and GABA (*Slc32a1*) (Fig. 1c).

Glial cells of the hypothalamus have been shown to play critical and tissue-specific roles in the regulation of osmolarity^32^, circadian rhythm^33^, metabolism^34^, and neurogenesis^35^. To better understand the molecular mechanisms controlling the specification and differentiation of hypothalamic glia, each glial population was reclustered and examined separately.

Cells that were identified as part of the oligodendrocyte maturation trajectory, and hence that share a similar molecular history, were reclustered as previously described^13,36^, and genes that demarcate each stage of oligodendrocyte development were identified with pseudotime^37^ and RNA velocity analysis^38^ (Supplementary Fig. 4a–c, Supplementary Data 1). To identify genes selectively enriched in hypothalamic oligodendrocytes, mature oligodendrocytes were directly compared to scRNA-seq datasets from mature cortical oligodendrocytes. While *Pcsk1n* and *Cbx3* are highly enriched in hypothalamic, relative to cortical, oligodendrocytes (Supplementary Fig. 4d–h), these genes are enriched in all hypothalamic glial cells, and are not specific to oligodendrocytes.

In contrast, we identified many genes that were both astrocyte-enriched relative to other glial cell types, and selectively expressed in hypothalamic, relative to cortical, astrocytes (Supplementary Fig. 5a–c). These include higher expression of *Agt*, and a lower level of *Mfge8* in hypothalamic astrocytes, as previously reported^36^, along with newly identified hypothalamic-enriched genes such as *Marcks* and *Marcks1* (Supplementary Figure 5a–c), which are important regulators of protein kinase C-dependent calmodulin signaling^39,40^. Analysis of the developing trajectory connecting non-neuronal gliogenic progenitor cells and hypothalamic astrocytes with RNA velocity and pseudotime analysis identified transitional states between these two populations. Immature hypothalamic astrocytes co-express the mature astrocyte marker *Agt*, and *Rgcc*, a cell-cycle regulator that regulates Notch signaling^41,42^ (Supplementary Fig. 5d–f, Supplementary Data 2). Loss of expression of genes specific to gliogenic progenitors was observed in hypothalamic astrocytes and other glial populations after the second postnatal week (Supplementary Fig. 5g). The upregulation of Notch signaling pathway components was also observed, as previously reported for human astrocyte development in vitro^43^ (Supplementary Fig. 5h).

Analysis of developmental trajectories for individual hypothalamic cell types identified the age at which these cell types began to diverge in gene expression, and identified both known and candidate regulators of cell fate. This is clearly seen when comparing the development of two ventricular glial-like cell populations—ependymal cells and tanycytes. These two classes of ventricular cells begin to diverge at E13, with differential expression of *Foxj1* and *Rax*—established markers of ependymal cells and tanycytes—first detected at this age (Fig. 1a, Supplementary Fig. 6)^44^. Pseudotime analysis paired with RNA velocity identifies additional transcription factors that are candidates for controlling tanycyte and ependymal cell specification and differentiation (Supplementary Fig. 6a, b, Supplementary Data 3). Transcription factors and morphogenic signaling components are highly enriched during the early stage of ependymal (i.e., *Wnt7b*, *Unc50*, *Vegfa*, *Ptch1*, *Nfib*, and Arxes2), and tanycyte (i.e., *Wnt7b*, *Ptch1*, *Notch3*, *Hopx*, and *Nr2f1*) development, and ependymal (i.e., *Rarres2* and *Foxj1*), and tanycyte (i.e., *Lhx2* and *Rax*) are at the strongest towards the end of pseudotime (Supplementary Fig. 6c, d).

### Profiling of region-specific genes in the developing diencephalon

**Fig. 2:**
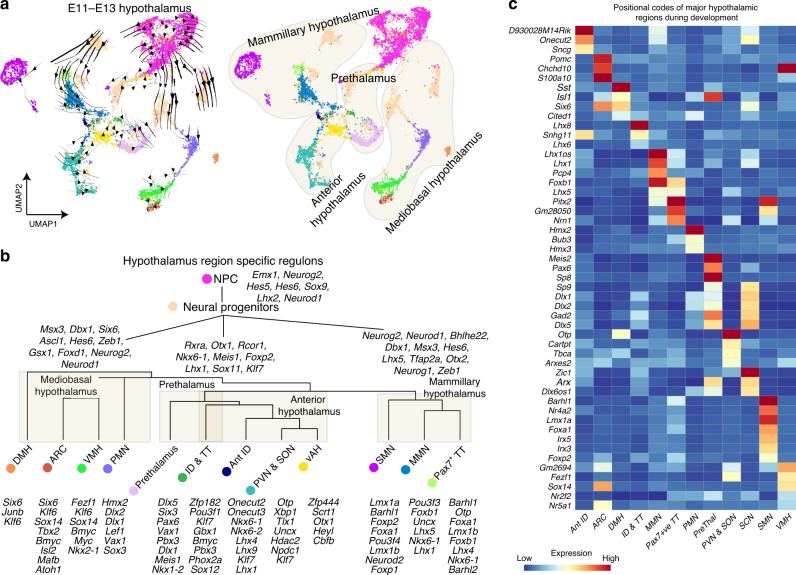
Specification of hypothalamic patterning during embryonic development. **a** UMAP plot showing E11–E13 developing diencephalon with RNA velocity trajectories (left), and UMAP plot showing the four main molecularly distinct regions of the developing hypothalamus and prethalamus (mediobasal hypothalamus, mammillary hypothalamus, anterior hypothalamus, and prethalamus) (right). **b** Dendrogram showing a developmental hierarchy of the entire diencephalon and associated regulons. **c** Heatmap showing a key subset of pattern-specific genes in major hypothalamic regions and prethalamus.

We next investigated whether we could use this dataset to faithfully distinguish hypothalamic domains that are spatially distinct in the embryo. To do this, we re-clustered data from E11 to E13, which correspond to the peak period of hypothalamic neurogenesis (Fig. 2a, Supplementary Fig. 7)^45^. Using previously identified region-specific markers as a reference^2^, we observed a clear segregation of spatially distinct neuronal precursors and progenitors (Fig. 2a, Supplementary Fig. 7). We were able to readily distinguish hypothalamic and adjacent cell populations including the prethalamus, discrete clusters for telencephalic structures such as preoptic area and medial ganglionic eminence, thalamic eminence, rim domain, and the main body of the sensory thalamus, as well as the zona limitans intrathalamica (ZLI) at all three developmental ages (Supplementary Fig. 7).

Each of the previously reported major subdivisions of the developing hypothalamus^2^ were also identified, including postmitotic neuronal precursor cells of the paraventricular nucleus/supraoptic nucleus (PVN/SON), extrahypothalamic diagonal (ID) and tuberomammillary terminal (TT), ventromedial hypothalamus (VMH), arcuate nucleus (ARC), premammillary hypothalamus (PMN), mammillary nucleus (MMN), and supramammillary nucleus (SMN) (Fig. 2b, Supplementary Fig. 7). In addition, several spatially distinct subtypes of mitotic hypothalamic progenitor cells were also observed, most notably cells that shared markers of both MMN and SMN (Supplementary Fig. 7, Supplementary Data 4). We also identified a clear separation between mitotic neural progenitors and postmitotic neural precursors (Fig. 2a, Supplementary Fig. 7).

RNA velocity paired with regulon analysis using SCENIC^46^ identified four main developmental trajectories, which give rise to mammillary hypothalamus, prethalamus, anterior hypothalamus, and mediobasal hypothalamus (Fig. 2a). Each of these developmental trajectories included multiple subdivisions of the developing hypothalamus. Hierarchical gene regulatory networks showed multiple regulons that are potentially involved in the differentiation of neural progenitors into neural precursors from the major spatial subdivisions of the developing hypothalamus (Fig. 2b).

Multiple known and previously undescribed molecular markers, including many transcription factors that act as positional codes and regulons, were further identified for each of these regions (Fig. 2c, Supplementary Data 4). While some of these markers are shared among multiple regions of the hypothalamus and other forebrain areas, others are highly specific and nonoverlapping. However, there was no substantial difference in expression patterns of neuropeptides and neurotransmitters across subdivisions of the developing hypothalamus that belong to the four main developmental trajectories (Supplementary Fig. 8), which could indicate that neuropeptides or neurotransmitter expression in each hypothalamic region are regulated by different gene regulatory networks. This analysis was able to efficiently identify gene expression patterns that were restricted to specific spatial domains and subdomains of the developing hypothalamus and prethalamus, confirming and extending our previous findings^2^.

Due to the high complexity of the hypothalamic clusters observed in both two- and three-dimensional analysis, it is difficult to comprehensively visualize region-specific differences in gene expression. To improve visualization of these data, we generated a heatmap for major pattern marker genes that corresponds to the two-dimensional sagittal plane, capturing the main spatial subdivisions of the developing hypothalamus and adjacent brain regions (Supplementary Fig. 9).

This analysis also identified clusters that correspond to three hypothalamic regions that had not been described in previous work^2^, including two populations of excitatory neurons. The first of these regions is found in the dorsomedial hypothalamus, and is marked by expression of *Sst*, *Cited1*, *Otp*, and *Six6* (Fig. 2c). The second region is found in the TT/PMN region, and expresses *Pax7* (Supplementary Data 4). The third region is found in the lateral hypothalamus (LH), and consists of a diverse collection of subtypes of neuronal precursors. This LH cluster consists primarily of glutamatergic neurons, with a small subpopulation of GABAergic neurons (Supplementary Fig. 10, Supplementary Data 6). The glutamatergic population includes a discrete subcluster of *Lhx9*-positive neurons, which marks precursors of hypocretin neurons^2,47,48^. Cells within this LH cluster express multiple transcription factors that are also selectively expressed in other hypothalamic regions, including the VMH, PMN, MMN and ID, as well as a mixture of neuropeptides expression (Supplementary Fig. 10).

Clustering of cells from previously characterized spatial domains of developing hypothalamus also identified discrete subclusters that express common sets of genes, as well as regulons that are enriched in the discrete subclusters of individual hypothalamic regions. This is clearly seen in the PVN/SON cluster (Supplementary Fig. 11, Supplementary Data 6). Selective expression of *Onecut2*, *Cartpt*, and *Zic1* characterizes a ventrolateral domain that, based on its position, likely corresponds to the developing SON (Supplementary Fig. 11).

This same approach can be readily applied to other forebrain regions. We have previously identified molecular markers that both identify discrete spatial domains within the prethalamus, which gives rise to structures such as the thalamic reticular nucleus and ventral lateral geniculate nucleus^2,49,50^, and investigated whether these regions could be identified using scRNA-seq data.

Subclustering of prethalamic cells allowed us to distinguish discrete spatial subdivisions within the prethalamus. We observed partially overlapping domains of expression of the transcription factors *Sp8* and *Sp9* (Supplementary Fig. 12, Supplementary Data 6), which play critical roles in the development of telencephalic interneurons^51^. We also identified a small ventral cluster that selectively expresses *Pbx1*. We also identified *Th* expression in the two prethalamic clusters which were enriched for the *Meis2*, but not the *Pax6*, regulon, indicating the *Meis2* might direct differentiation of dopaminergic neurons^52^. ISH analysis revealed enriched expression of *Sp8* in anterior prethalamus and ID, while *Sp9* was enriched in posterior prethalamus (Supplementary Fig. 12).

Subclustering of the VMH allowed us to detect two distinct clusters, which corresponded to separate anterior and posterior domains of gene expression (Supplementary Fig. 13, Supplementary Data 6). A clear distinction between these anterior and posterior domains was detected until E16, both spatially and at the molecular level (Supplementary Fig. 13). These two clusters had begun to spatially intermingle, yet the molecular distinction still remained, possibly reflecting local tangential cell migration within the VMH.

By combining our analysis of both the molecular markers of differentiation of major hypothalamic cell types and the selective markers of the different spatial domains of the developing hypothalamus and prethalamus, we have compiled a reference set of molecular markers that will be useful for further functional studies. We have designated this integrated and annotated scRNA-seq dataset as HyDD, or the *Hy*pothalamus *D*evelopmental *D*atabase.

### HyDD identifies developmental origins of VMH neurons

**Fig. 3:**
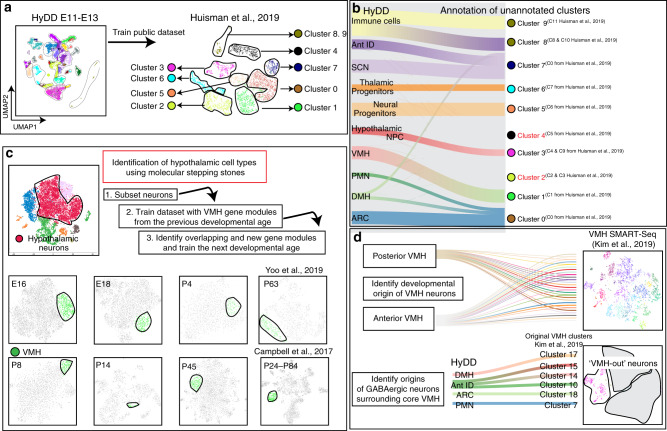
Utilizing HyDD to infer the identity and origin of individual cell types. **a** The HyDD dataset was used to train a previously published scRNA-seq on E15.5 hypothalamus obtained through selective dissection of *Pomc-EGFP*-expressing cells^53^. **b** Alluvial plot showing HyDD clusters (left) matched to clusters from Huisman et al.^53^ (right). Note that 2 clusters (clusters 2 and 4) from Huisman et al.^53^ do not match the HyDD dataset. **c** Using the molecular stepping stone approach to identify VMH neurons (green) across the entire developmental ages by identification of shared sets of gene modules that can demarcate the VMH across the entire hypothalamus scRNA-seq dataset. **d** HyDD dataset is used to identify the developmental origins of previously annotated subtypes of glutamatergic neurons of the core VMH^55^ (top), and to identify the developmental origins of GABAergic neurons surrounding the core VMH (bottom).

To demonstrate the broad usefulness of the HyDD, we first annotated a previously published scRNA-seq dataset obtained through selective dissection of *Pomc-EGFP*-expressing cells from E15.5 hypothalamus using regional and cell type-specific markers from the HyDD^53^. In this study, while one cluster (cluster 0) was previously identified as the developing ARC, the remaining clusters were not annotated owing to the lack of well defined regional and cell type-specific markers to resolve the spatial location of these clusters. Using markers obtained from the HyDD to train the dataset, we were able to annotate all but two clusters, representing cells from multiple hypothalamic regions, including VMH, PMH, anterior ID, DMH, SCN, and ARC. Some clusters were composed of cells from multiple hypothalamic regions, which may explain some of the previous difficulties in annotating these cells (Fig. 3a, b). Two unannotated clusters appear to reflect contamination from the habenula and pituitary that occurred during dissection (Supplementary Fig. 14). A subset of the neurons in the ARC cluster share molecular markers of neural precursors in the PMN and DMH, implying that these cells may have migrated to the ARC from these regions (Supplementary Fig. 14).

We also identified a cluster that closely resembled hypothalamic NPC (Supplementary Fig. 15), but which also co-expressed astrocyte-, ependymal, and/or tanycyte-specific marker genes. Gene sets enriched in this cluster were then projected into the entire hypothalamus scRNA-seq dataset (E10–P45), and glial populations including immature glial cells were enriched with these gene sets. This same gene expression pattern was found to be enriched in a subset of hypothalamic NPC that were detected from E11 onwards, and which may represent NPC that are competent to generate glia (Supplementary Fig. 15). Many of these same genes are also expressed in the late-stage retinal progenitor cells, from the age at which they become competent to give rise to tanycyte-like Mũller glial cells^31^.

Since HyDD contains a nearly uninterrupted temporal profile of changes in gene expression during the process of cell specification and differentiation, it can also be used to infer the developmental origins of fully mature hypothalamic neurons. However, identifying the precise spatial location of individual cell types from hypothalamus scRNA-seq data based on specific molecular markers alone is bioinformatically challenging, due to the extreme tissue complexity. This is the case even when scRNA-seq data has been generated with microdissected or flow-sorted cells from predefined hypothalamic regions. Most informative region-specific markers are strongly expressed early in development, but are either not expressed or show substantially different expression levels at later developmental ages^2^. Postmitotic hypothalamic neural precursors also undergo a considerable amount of tangential migration and dispersion, making it even harder to directly identify gene regulatory networks that control the specification of individual hypothalamic cell types^54^.

To identify the developmental origin of individual hypothalamic cell types, it is critical that overlapping sets of markers be identified that selectively label each stage of cell differentiation, in a manner analogous to molecular stepping stones, so that the developmental history of each cell type can be reconstructed. As a proof of principle for this approach, we identified gene sets that identify VMH cells at early stages of hypothalamic development (Fig. 3c), when region-specific molecular markers are robustly expressed. Gene sets specific to discrete spatial domains were then used to train the following developmental age to find VMH cells and new VMH-enriched genes were identified. This process was repeated for each successive developmental age. These VMH-enriched genes have varying levels of expression and specificity across the full course of the hypothalamus development (Supplementary Fig. 16).

We next used the HyDD to identify the developmental origin of major VMH neuronal subtypes. Recent scRNA-seq of the adult VMH identified multiple clusters of both core glutamatergic VMH neurons and of GABAergic neurons surrounding the core VMH (VMH-out)^55^. We sought to identify the developmental origins of both classes of VMH neurons. We first found that GABAergic neurons of VMH-out originated from four distinct regions of the developing hypothalamus - ARC, DMH, Ant ID and PMN (Fig. 3d)—with each VMH-out GABAergic cluster having a distinct developmental origin based on the specific expression of regional markers. We likewise observed that different subsets of core glutamatergic VMH neurons arise from distinct anterior or posterior domains of the embryonic VMH (Supplementary Fig. 13). Some of these clusters remain restricted to anterior or posterior regions of the adult VMH, as noted in the original study^55^ (Fig. 3d, Supplementary Fig. 17a, b). However, the majority of VMH neuronal subtypes originate from both anterior and posterior domains of the developing VMH (Supplementary Fig. 17), and are distributed widely along the anterior–posterior axis of the adult VMH^55^. VMH neuronal subtypes may thus be two distinct developmental steps: an initial stage in which anterior and posterior identity is specified between E11 and E13, and a later stage that coincides with the initiation of local tangential migration that occurs from E16 onwards.

### HyDD allows comprehensive analysis of complex mutant phenotypes

HyDD provides both a high-resolution molecular atlas of the developing hypothalamus and prethalamus, and a useful resource to understand the developmental origin of adult hypothalamic neurons. We next sought to determine if HyDD could also be used to rapidly and comprehensively characterize mutants that regulate early stages of hypothalamic development and organization. As proof of concept, we performed scRNA-seq analysis on E12.5 *Foxd1*^*CreGFP/+*^*;Ctnnb1*^*ex3/+*^ mice, in which a constitutively active form of beta-catenin is overexpressed in *Foxd1*-positive hypothalamic and prethalamic progenitors, leading to activation of canonical Wnt signaling in these cells and their descendants^23^. The same analysis was also with *Foxd1*^*CreGFP/+*^ littermate controls. These mice show broad activation of the canonical Wnt pathway effector *Lef1*, a hyperplastic ventricular zone, and with the exception of a handful of posterior hypothalamic markers, show the loss of most regional markers in the hypothalamus and prethalamus^23^.

ScRNA-seq analysis of control and mutant animals at E12.5 reveals a couple of mutant-specific cell clusters (Supplementary Figs. 18–20). Using the HyDD to annotate both control and mutant data, we identified changes in gene expression and cell composition that match previously reported findings (Supplementary Fig. 20), where we observed a substantial increase in undifferentiated NPC, along with a corresponding reduction in the number of cells expressing markers of hypothalamic and prethalamic neuronal precursors (Supplementary Figs. 19 and 20). RNA velocity further highlights differences in developmental trajectories between control and mutants (Supplementary Fig. 20). In particular, strong loss of markers shared by both hypothalamus and prethalamus, such as *Meis2*, *Sp9*, and *Arx* (Supplementary Fig. 20, Supplementary Data 7) was observed. We also identified two cell clusters that are found exclusively in mutant mice, both of which express NPC markers, and also highly express both *Lef1* and negative regulators of canonical Wnt signaling such as *Dkk1*, *Wif1*, and *Axin2* (Supplementary Fig. 18e). One of these clusters is strongly enriched for G2/M phase markers such *Ube2c*, *Rrm2*, and *Ccnb1* (Supplementary Figure 19). Flow cytometry data also demonstrated a substantially higher fraction of NPCs in the G2/M phase in mutant mice (Supplementary Figs. 19 and 20), as has been previously reported in nonneuronal cells that show high levels of canonical Wnt signaling^56^. This finding explains the previous observation that, although a massive increase in the number of NPC cells is seen in these mutants, only a modest increase is observed in EdU labeling, which labels S-phase NPC^23^. This demonstrates the power of using scRNA-seq in conjunction with the HyDD to analyze developmental phenotypes, in a manner that is far more rapid and comprehensive than conventional histological techniques.

We next used this same approach to characterize E12.5 *Nkx2-1*^*CreER/CreER*^ knockin mice, which are homozygous for a null allele in the homeodomain transcription factor *Nkx2-1*^57^. *Nkx2-1* is broadly and selectively expressed in ventral hypothalamic progenitors, as well as in progenitors that give rise to telencephalic interneurons^58,59^. Loss of function of *Nkx2-1* leads to a substantial reduction in ventral hypothalamic structures by E18^60^, but a detailed molecular characterization of these mutants has not been conducted.

**Fig. 4:**
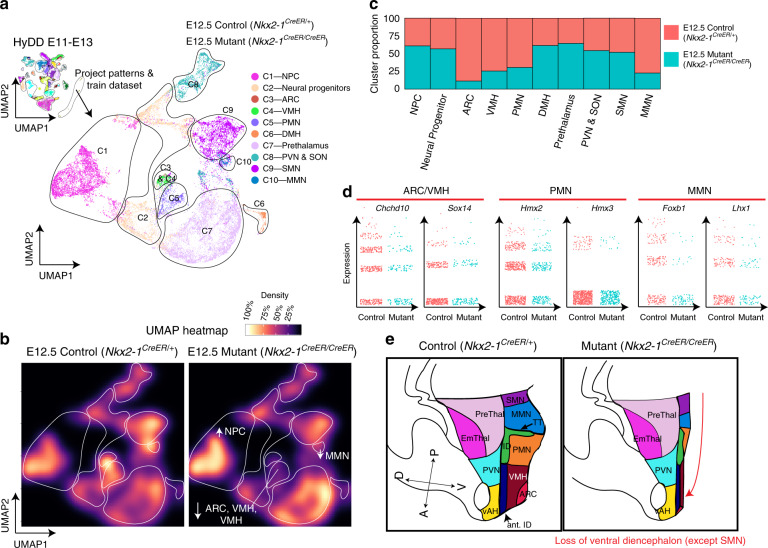
scRNA-seq-based analysis of *Nkx2-1*-deficient developing hypothalamus. **a** UMAP plot showing clusters from combined scRNA-seq dataset of control (*Nkx2-1*^*CreER/+*^) and *Nkx2-1* mutant line (*Nkx2-1*^*CreER/CreER*^), in which clusters were obtained by training the dataset using HyDD markers. **b** UMAP heatmap plot showing distribution of individual clusters between control (left) and *Nkx2-1* mutants (right). **c** Bar graph showing the distribution of individual clusters between control and *Nkx2-1* mutants. **d** Jitter plots of regional marker genes (*Chchd10* and *Sox14*—ARC/VMH, *Hmx2* and *Hmx3*—PMN, *Foxb1* and *Lhx1*—MMN between control and *Nkx2-1* mutants. **e** Schematic showing overall hypothalamic phenotype between control and *Nkx2-1* mutants. Note the absence of ventral diencephalic structures (except the supramammillary nucleus), and the relative expansion of the prethalamus in *Nkx2-1* mutants (right).

Analysis of *Nkx2-1*^*CreER/CreER*^ mutants and heterozygous littermate controls revealed changes in cluster densities in the mutant (Fig. 4, Supplementary Figs. 21 and 22). We observed a broad loss of markers specific to *Nkx2-1* positive ventral hypothalamic structures such as ARC, VMH, PMN, and MMN, but not the SMN (Fig. 4c, d, Supplementary Figs. 23 and 24, Supplementary Data 8), with both the relative expression levels and the number of cells expressing these markers reduced. An increase in the fraction of cells expressing prethalamic markers was detected (Fig. 4b, c, Supplementary Fig. 21), and increased Cre expression in the prethalamus was also observed in these mice (Supplementary Fig. 22).

In contrast to controls, prethalamic cells in mutant mice expressed *Cre*, implying that ventral hypothalamic cells that normally express *Nkx2.1* may have acquired prethalamic identity (Supplementary Fig. 22). This was also implied with an increase in cells in the prethalamic cluster coupled with a decrease in cells ventral hypothalamus (mediobasal and mammillary excluding SMN), but no substantial overall change in the portion of neural progenitors (Fig. 4b, c). Furthermore, our RNA velocity analysis strongly indicates altered trajectories between mediobasal hypothalamus, mammillary hypothalamus, and prethalamus (Supplementary Fig. 22.) To investigate this further, RNAscope probes against *Sp9*, *Meis2*, and *Cre* were used to visualize the location of these *Cre*-positive prethalamic cells, and substantial co-localization of prethalamic markers and *Cre* expression was observed in the region normally occupied the by the ventral hypothalamus in controls (Supplementary Fig. 22). This implies that *Nkx2-1* not only maintains the identity of ventral hypothalamic progenitors but also actively represses the expression of molecular markers of prethalamic identity. ISH confirmed that there was an increase in the absolute size of the prethalamus and its proportion in the diencephalon (Supplementary Fig. 23). An increase in the number of cells expressing markers of NPC in the SMN and MMN was also seen, while *Nkx2-1* negative hypothalamic regions such as the PVN/SON are unaffected (Fig. 4d, Supplementary Fig. 24).

There was no significant difference in gene expressions between *Cre*+ and *Cre*− pre thalamic cells (Supplementary Fig. 22), but using regulon analysis, mutant prethalamus showed enriched activity for transcription factors that are enriched in the mediobasal hypothalamus, such as *Foxp1*, *Six3*, and *Zfhx3* (Supplementary Fig. 25).

## Discussion

In this study, we use scRNA-seq to develop a molecular atlas of the developing mouse hypothalamus, with a particular focus on stages when hypothalamic patterning and neurogenesis are regulated. This dataset identifies genes that are selectively expressed during the differentiation of major neuronal and nonneuronal hypothalamic cell types, and accurately delineates spatial subdivisions present in the early stages of development of both the hypothalamus and the adjacent prethalamus. It also identifies many previously uncharacterized transcription factors and other genes that are excellent candidates for controlling regional patterning and specification of individual hypothalamic cell types. Combining functional analysis of these genes with the new selective markers of hypothalamic regions and immature hypothalamic cell types identified in this study has the promise to greatly expand our knowledge of hypothalamic development and organization.

The integrated dataset presented here provides three specific features that are critical for studying the formation and function of the hypothalamus. First, it makes it straightforward to unambiguously annotate major cell types at all stages of hypothalamic development. Second, it makes it possible in many cases to infer the developmental histories of hypothalamic cells in both the developing and mature hypothalamus. Third, it allows rapid and accurate phenotyping of mutants that show broad effects on hypothalamic patterning, neurogenesis, and differentiation, with which we were able to validate our findings using traditional histological analysis. Despite the availability of highly specific molecular markers for the major spatial subdivisions of the hypothalamus^15^, the highly complex and temporally dynamic anatomy of this brain region makes analysis of mutant phenotypes slow and complex. Previously, it has taken up to several years of full-time labor to obtaina detailed characterization of individual mutant lines. The HyDD dataset allows these analyses to be conducted far more rapidly, efficiently, and comprehensively.

Our scRNA-seq characterization of *Nkx2-1*-deficient mice identifies an unexpected developmental connection between the hypothalamus and prethalamus, where *Nkx2-1* can potentially act as both a positive regulator of ventral hypothalamic identity while simultaneously repressing prethalamic identity. This result is not predicted by the current prosomeric model for forebrain organization^61,62^, and raises questions about the early development and patterning of these structures. Previous models of hypothalamic development and organization were constructed using very sparse datasets—typically single color ISH of a limited number of genes at a small number of time points. The much richer datasets provided by scRNA-seq, and interpreted usin the HyDD data, offer a far more powerful resource for constructing these models.

## Methods

### Mice

All experimental animal procedures were approved by the Johns Hopkins University Institutional Animal Care and Use Committee. All mice were housed in a climate-controlled facility (14-h dark and 10-h light cycle) with ad libitum access to food and water. Time-mated CD1 or C57BL/6J mice were ordered from Charles River Laboratories to collect mice embryos at E10 (CD1), E11 (CD1), E12 (CD1), E13 (CD1), E14 (CD1), E15 (CD1), E16 (1 CD1 and 1 C56BL/6J), and E18 (CD1). Time-mated CD1 mice were ordered to collect pups at P4, P8, and P14. C57BL/6J mice were used for P45 samples.

*Nkx2-1*^*CreERT2*^ knockin^57^ (JAX #014552), *Foxd1*^*CreGFP*^ knockin^63^ (JAX #012463), *Ctnnb1*^*ex3/ex3* 64^ were used for single-cell phenotyping studies. *Ctnnb1*^*ex3/ex3*^ mice were crossed with *Foxd1*^*CreGFP/+*^ to generate *Foxd1*^*CreGFP/+*^ or *Foxd1*^*CreGFP/+;*^*Ctnnb1*^*ex3/+*^. *Nkx2-1*^*CreERT2*^ was C56BL/6J background, and *Foxd1*^*CreGFP/+*^ knockin, *Ctnnb1*^*ex3/ex3*^ were C57BL/6 and CD1 mixed background. Mice were time-mated during their estrous cycle and vagial plugs were observed to detect successful mating. E12.5 embryos were collected for generating scRNA-seq dataset.

### Dissection and cell dissociation

Embryos or postnatal mice were collected and dissociated using a previously published protocol^65^. Embryos were collected using Hibernate-E media (Thermo Fisher Scientific) with 2% B-27 supplement (Thermo Fisher Scientific) and GlutaMAX supplement (0.5 mM final, Thermo Fisher Scientific). A small incision was made dorsal to the lower jaw to expose the ventral portion of the brain. For samples collected between E10 and E16, tissue residing posterior to the medial ganglionic eminence and anterior to the midbrain and sensory thalamus was dissected to collect both developing prethalamus and hypothalamus. Prethalamus was excluded from samples aged E18 and older, with only hypothalamus collected, as previously described^2^. Exclusion of medial ganglionic eminence (anterior to the hypothalamus) and structures posterior to the supramammillary nucleus ensured that the equivalent diencephalon area (hypothalamus and prethalamus) was always included. Between 8 and 12 embryos of either sex were collected for each embryonic time point and pooled for scRNA-seq dataset.

Postnatal mice were collected using Hibernate-A media with 2% B-27 and GlutaMAX (0.5 mM final), and the region that is posterior to the optic chiasm (Bregma −0.58 mm) and anterior to the hypothalamus–midbrain border (Bregma 2.54 mm) were collected. Eight pups (four male and four female) were collected for P4, P8, and P14 dataset, and three male mice were pooled for P45 dataset. E10, E12, and E15; E11 and E13; E14, E16, and P45; E18, P4, and P14 were each generated on the same day. Consecutive developmental ages were not collected and processed on the same day and this ensured that trajectories identified using tSNE and UMAP are not determined by batch-effect but by developmental stages.

For single-cell phenotyping studies, E12.5 time-mated embryos were collected and placed in a buffer mentioned above on ice. Tail-tips were collected and rapidly genotyped using GeneAmp Fast PCR master mix (Thermo Fisher Scientific). Both control and mutant groups were collected on the same day, and E12.5 embryos were pooled from three different dams. Between six and eight embryos were collected to generate individual scRNA-seq libraries.

Following dissection, tissues were dissociated in papain (Worthington Biochemical) as previously described in calcium-free Hibernate media^65^. Tissue debris were removed using OptiPrep density gradient media (Sigma-Aldrich) in postnatal mice following cell dissociation. Numbers of viable cells were counted manually via haemocytometer with Trypan Blue staining and cross-checked with an automated cell counter, and cell concentration was adjusted following the manufacturer’s protocol of 10× Genomics.

### ScRNA-seq library generation and data processing

Suspended cells were loaded into 10× Genomics Chromium Single Cell System (10× Genomics), and libraries were generated using v1 (1 library) and v2 chemistry with manufacturer’s instructions. Libraries were sequenced on Illumina MiSeq (1 library) and NextSeq500 high-output (400 million reads). Sequencing data were first pre-processed through the Cell Ranger pipeline (10× Genomics, Cellranger count v2.0.2) with default parameters (expect-cells set to number of cells added to 10× system), aligned to mm10 genome (refdata-cellranger-mm10-1.2.0), and matrix files were used for subsequent bioinformatic analysis.

### Data analysis

For analysis of the entire hypothalamic dataset, Seurat v3.10^66,67^ and Scanpy v1.51^68^ were used to process matrix files to keep all cells with at least 200 detected genes and 500 UMI. Datasets were normalised using Seurat “scTransform” function, and Harmony v1.0^69^ was used when treating individual scRNA-seq (orig.ident) as a variance group, to reduce inter-batch effect and adjust for individual variation.

For the entire hypothalamic dataset, the top 2000 highly variable genes were used for principal component analysis (PCA), and top 50 PCA variables were used for either UMAP, to preserve global distances to better visualise changes across developmental stages^70^.

Individual ages were initially clustered using the Louvain clustering algorithm provided in Scanpy with default parameters. Individual developmental ages were highlighted in the UMAP dataset, and individual cell types (or hypothalamic regions) were projected into UMAP to determine segregation of cell types (or regional markers) in the dataset regardless of developmental ages (https://proteinpaint.stjude.org/F/mm10/example.scrna.html).

For neuronal clusters, clustering was conducted until no child clusters showed any differential gene expression in neuropeptide/neurotransmitter/positional code (transcription factor) expression. The spatial location of each neuronal cluster was identified based on transcription factor positional codes identified from HyDD analysis in combination with Allen Brain in situ atlas data analyzed using co-coframer^22^.

To identify region-specific differences between mature oligodendrocytes or astrocytes of the hypothalamus and other brain regions, previously published cortical scRNA-seq^36,71^ were used to identify differential gene expression, using age as the variance with default parameters (first-pass gene lists)^37^. Published hypothalamic datasets^11,14,36,72^ were then used to compare to these cortical scRNA-seq datasets using the identified genes (second-pass gene lists). Identified differential genes were then validated by matching the Allen Brain in situ atlas data using co-coframer^11^ to validate spatial expression of these differential genes, as some observed differences could reflect batch effects resulting from scRNA-seq library preparations from different laboratories (third-pass gene lists).

To identify the developmental origins of ependymal cells and subtypes of tanycytes, transcription factors that are highly expressed near the midpoint of the pseudotime branch—where cells are not full mature tanycytes but no longer gliogenic progenitors—were extracted and clustered to scRNA-seq data obtained from adult tanycytes^14,73^ using Garnett v0.2.9^74^.

For analysis of hypothalamic patterning and generation of the developmental database HyDD (Hypothalamus Developmental Database), the E11–E13 datasets were used to perform a detailed analysis of hypothalamic patterning^2^, and processed as described above using the top 2000 highly variable genes with top 30 PCA variables. Initial clustering was conducted using the Louvain clustering algorithm in Scanpy with 0.6 resolution^75^, and individual clusters (initially divided based on rough anatomical locations and by cell cycle status) were further subdivided to capture all the main subdivisions of the developing hypothalamus, prethalamus, and adjacent structures. The initial clustering results were then cross-referenced to patterns from scCoGAPS, which were superior in capturing changes in gene expression over time, and selective markers of small sub-regions of the developing hypothalamus and prethalamus. A dendrogram showing hierarchical regulation of hypothalamus development was generated based on RNA velocity trajectories and positional codes to divide into four regions (mediobasal hypothalamus, prethalamus, anterior hypothalamus, and mammillary hypothalamus), and a distance matrix used in PCA space for the branch specification.

Cross-referencing between these two pipelines allowed us to identify all the sub-regions of the developing hypothalamus, prethalamus, and adjacent structures. Following subdivisions, enriched genes (top 50 highly enriched genes in an individual cluster) in the individual cluster were extracted and cross-referenced back to our previous work^2^, ISH validation, Allen Brain in situ atlas^21,22^ and GenePaint to further validate our cluster assignments^21,22^, as well as identifying pattern-specific markers. The final main clusters were subsetted and reclustered to identify additional subdivisions within the major hypothalamic and prethalamic regions. This was repeated until the child clusters could not generate any further clusters that had differential expression of transcription factors or no visible spatial distinction between markers under the Allen Brain in situ atlas.

To identify developmental hypothalamic clusters expressing neuropeptide markers, a list of neuropeptides from ref. ^17^ was used to identify neuropeptides that are expressed in a neuronal subtype-specific manner, and that show an increased expression (or an increase in percentage of cells expressing) among clusters between E11 and E13.

For clustering previously published datasets^76^, data were processed as previously described in the original paper. The HyDD dataset was used as the reference point to train the dataset using Garnett, using the top ten most selective markers identified from individual HyDD clusters, and identified clusters that were aligned to our dataset. For unannotated or poorly annotated clusters (which comprised less than 5% of all individual cells trained by our dataset), markers were identified and checked using the Allen Brain in situ atlas. These clusters were located outside of developing hypothalamus, in the habenula and pituitary, further validating the accuracy of the molecular markers identified in this study. An alluvial plot was then generated based on the percentage of clusters that were trained using our HyDD dataset. The opposite approach was taken to cross-validate HyDD annotation and to identify NPC that express high levels of gliogenic gene sets.

To identify VMH cells across the entire course of hypothalamic development using the molecular stepping stone approach, gene sets identified as labeling the VMH from the E11 to E13 datasets used to generate HyDD (markers that are significantly expressed in the VMH but not in ARC, Supplementary Data 4), were used to train the next developmental age (E14) and identify VMH from E14 scRNA-seq dataset using Garnett^74^. Following the identification of the VMH, scCoGAPS was used to identify a VMH-specific gene expression pattern (i.e., the set of expressed genes that can selectively demarcate the VMH), and identified new and overlapping gene sets of VMH cells. These gene sets were then used to train the next developmental age (E15), and this process was repeated to the oldest age (P45) in our scRNA-seq dataset. Genes that could identify the VMH across at least three developmental ages, or at least two developmental ages if the gene in question could also selectively specific cell clusters in the adult VMH, were selected as the final VMH gene sets (Supplementary Fig. 16). This process was necessary to identify cells comprising specific hypothalamic nuclei, since at older ages, genes that are highly informative at providing information about spatial localization within the hypothalamus are often no longer highly expressed. This molecular stepping stone approach was validated by identification of glutamatergic VMH neurons using the public droplet-based scRNA-seq dataset^14,73^. Spatial information of the identified clusters were validated by comparison to the Allen *Brain* in situ atlas data using co-coframer^22^, as well as by matching to the published SMART-seq data obtained from the adult VMH^55^.

To identify the developmental origins of GABAergic neurons surrounding the core VMH (VMH-out), clusters obtained from public SMART-seq data capturing only VMH^55^, were generated to match the previously published clusters. VMH-out GABAergic cells were then trained using HyDD region-specific markers, as expression of many region-specific markers persisted into the adult stage (although usually at low levels), and could readily be detected by SMART-seq.

To identify the developmental origin of major cell clusters in the VMH, key gene sets from the anterior and posterior VMH were extracted from HyDD (Supplementary Fig. 13, Supplementary Data 6). These gene sets of the anterior and posterior VMH were validated in both our dataset at later developmental ages and in a published dataset using the molecular stepping stone approach^53^. Additional histological validation was conducted using RNAscope (described below) and using the Allen Bran in situ atlas, anterior and posterior VMH gene sets were used to train VMH clusters. Some clusters clearly labeled either anterior or posterior domains of the embryonic VMH, and were also restricted to the corresponding region of the adult VMH^55^, validating this approach.

For mutant phenotyping, both control and mutants of individual lines were first merged together using the above method. Given the complex phenotypes of these mutants, we used sets of region-specific markers obtained from HyDD, we trained each individual dataset using Garnett^74^, which allowed us to faithfully cluster the mutant dataset, since the majority of pattern-specific markers were expressed in both genotypes, although often at different cellular expression levels. This approach allowed us to identify clusters that did not match annotated regions in HyDD, indicating the presence of mutant-specific clusters. Gene expression differences between control and mutants were compared between each identified region of the developing hypothalamus and prethalamus. For unidentified regions in mutant samples, gene expression was compared to all control regions. The percentage of regions occupied by cells of either genotype was compared as well. Both changes in pattern-specific markers and percentage of clusters occupied by each genotype reflect biological differences, but not changes in gene expression that resulted from variation in conditions occurred during dissection and library preparation. Top differential pattern-specific markers (mostly transcription factors) in regions that were differentially occupied between control and mutants were then selected for histological validation. *xy* coordinates from tSNE were used to estimate density (contour heatmap) of individual clusters between control and mutant groups.

Differential gene tests on the scRNA-seq datasets were initially performed using Seurat v3.1.0 FindAllMarkers using MAST with default parameters on all expressed genes by using individual dataset (age or genotype) as a variance, and cross-referenced to Monocle2 VGAM likelihood ratio tests using age or genotype as the full model were then used in differential expression tests with default parameters.

### scCoGAPS analysis

scCoGAPS (v.3.7.0), a Bayesian nonnegative matrix factorization algorithm, was used to identify “patterns”, or sets of co-expressed genes and their cellular expression levels, their weights in either the E11–E13 dataset or the individual/combined E10–P45 dataset, in order to identify cells that represent gliogenic progenitors or immature glia, or to identify VMH at individual developmental ages, using previously described parameters^77^.

All genes were used to identify patterns for E11–E13 dataset, scCoGAPS patterns were projected into our tSNE plot and interpreted by using our prior knowledge of gene expression patterns in the developing hypothalamus. For the molecular stepping stone approach used to trace the developmental history of VMH neurons, or for identifying gliogenic progenitor populations. 200 HyDD cell or region-specific markers were used to annotate patterns, with the ‘patternMarkers’ function in order to identify robust pattern markers across developmental stages.

### E12 spatial mapping

*xy* coordinates of E12 were drawn based on our previous work to capture all postmitotic regions of the developing hypothalamus and prethalamus, average values of individual clusters were assigned to each *xy* coordinate, and 2D spatial representation was produced using the Python-based SpatialDE package v1.1.3^78,79^.

### Cell cycle analysis

Cell cycle data for NPCs from *Foxd1*^*Cre/+*^;*Ctnnb1*^*Ex3/+*^ mice overexpressing constitutively active *Ctnnb1* in hypothalamus and prethalamus, as well as age-matched controls, were analyzed using scran v3.11^76^. Additional cell cycle analysis was conducted by staining dissociated E12.5 control and mutant developing diencephalon with propidium iodide and analysed DNA content using LSR II (BD) and FlowJo v10.6.1.

### RNA velocity

RNA velocity38 was utilized to understand the dynamic state of the entire state of hypothalamic development, gliogenesis (oligodendrocytes, astrocytes, and ependymal and tanycyte development), during when complex patterning occurs across multiple hypothalamic regions, and to identify potential differences in developmental trajectories between control and mutant samples. Kallisto and bustools^80,81^ python wrapper kb-python was used to obtain spliced and unspliced transcripts using -lamanno with GRCm38 mouse genome. Scanpy^68^ and scVelo v0.2.1^82^ was used to process the Kallisto output with default parameters, based on UMAP coordinates obtained from Seurat.

### Pseudotime analysis

Monocle3 v0.2.0^37^ was used to perform pseudotime analysis to identify differences in gene expression in differentiating oligodendrocytes, astrocytes, and tanycytes and ependymal cells, where the principal and trajectory nodes were identified based on trajectories from RNA velocity analysis, and genes were used for pseudotime plotting was identified based on high-variance genes on the order of cells in pseudotime with *q* value less than 0.001.

### Regulons

To identify regulons controlling gene expression in different hypothalamic regions during hypothalamus patterning, SCENIC^46^ using python implemented pySCENIC v0.10.2 Gene regulatory networks (uising -masks_dropouts), regulons and network activity of regulons were calculated using default parameters with mm10 feather files on E11–E13 scRNA-seq dataset using raw count matrix. Regulon specificity scores were ranked following the SCENIC pipeline and top regulons with *z*-score higher than two were identified as the cluster/cell-type regulon.

### In situ hybridization (ISH)

Chromogenic ISH was performed as previously described^2^, except E12.5 or E13.5 embryos were fixed with 4% paraformaldehyde, sectioned at 25 µm with either coronal or sagittal plane and treated with proteinase K for 5 min at room temperature. *Cartpt* (BC056431), *Zic1* (AI848240), *Zic3* (BF465672), *Arx* (BE944865), Dlx5 (AW046057), *Sox14* (BU517725), *Foxb1* (BC111908), *Hmx2* (BC023402), and *Foxa1* (BC096524) were used.

Single-molecule fluorescence ISH was performed using RNAScope with probes targeting *Meis2*, *Sp8*, *Sp9*, *Pitx2*, *Nhlh2*, *Tcf7l2*, *Foxp2*, *Snca*, and *Cre* on E12.5 or E13.5 wild-type or *Nkx2-1*^*CreERT2/CreERT2*^ embryos; and *Nhlh2*, *Foxp2, Snca* on E13.5, E16.5, E18.5, and P4 wild-type mice following the manufacturer’s protocol.

### Immunostaining

Fixed embryos were processed for immunostaining with Pax6-antibody (1:200, AB2237, EMN Millipore). Sections were mounted with Vectamount (Vectorlabs) and imaged under Keyence BZ-X800 fluorescence microscope and Zeiss LSM 700 microscope.

### Statistics and reproducibility

All staining was performed in triplicates, with tissues from at least two different litters.

### Reporting summary

Further information on research design is available in the Nature Research Reporting Summary linked to this article.

## Supplementary information

Supplementary Information

Description of Additional Supplementary Files

Supplementary Data 1

Supplementary Data 2

Supplementary Data 3

Supplementary Data 4

Supplementary Data 5

Supplementary Data 6

Supplementary Data 7

Supplementary Data 8

Supplementary Data 9

Reporting Summary

## Data Availability

Data supporting the findings of this work are available within the paper and its Supplementary Information files. All scRNA-seq data are available on GEO, GSE132355. Data can be viewed at https://proteinpaint.stjude.org/F/mm10/example.scrna.html.
