# Author Correction: The cellular and molecular landscape of hypothalamic patterning and differentiation from embryonic to late postnatal development

**DOI:** 10.1038/s41467-021-27676-9

**Published:** 2022-01-11

**Authors:** Dong Won Kim, Parris Whitney Washington, Zoe Qianyi Wang, Sonia Hao Lin, Changyu Sun, Basma Taleb Ismail, Hong Wang, Lizhi Jiang, Seth Blackshaw

**Affiliations:** 1 Solomon H. Snyder Department of Neuroscience, Johns Hopkins University School of Medicine, Baltimore, MD 21205 USA; 2 Department of Ophthalmology, Johns Hopkins University School of Medicine, Baltimore, MD 21205 USA; 3 Department of Neurology, Johns Hopkins University School of Medicine, Baltimore, MD 21205 USA; 4 Center for Human Systems Biology, Johns Hopkins University School of Medicine, Baltimore, MD 21205 USA; 5 Institute for Cell Engineering, Johns Hopkins University School of Medicine, Baltimore, MD 21205 USA; 6 Kavli Neuroscience Discovery Institute, Johns Hopkins University School of Medicine, Baltimore, MD 21205 USA

**Keywords:** Cell fate and cell lineage, Neural patterning, Neural progenitors

Correction to: *Nature Communications* 10.1038/s41467-020-18231-z, published online 31 August 2020.

The original version of this Article contained an error in ‘Data analysis’, which incorrectly read: ‘For analysis of the entire hypothalamic dataset, Seurat v3.10 and Scanpy v1.5 were used to process matrix files to keep all cells with at least 200 detected genes’.

The correct version adds ‘and 500 UMI’ after ‘200 detected genes’. This has been corrected in both the PDF and HTML versions of the Article.

The original version of this Article contained an error in ‘Data analysis’, which incorrectly read: ‘Datasets were normalised using Seurat ‘scTransform’ function, and Harmony v1.0 was used when treating individual scRNA-Seq as a variance group, to reduce inter-batch effect and adjust for individual variation’.

The correct version adds ‘(orig.ident)’ after ‘when treating individual scRNA-Seq’. This has been corrected in both the PDF and HTML versions of the Article.

The original version of this Article contained an error in ‘Data analysis’, which incorrectly read: ‘For the entire hypothalamic dataset, the top 2000 highly variable genes were used for Principal Component Analysis (PCA), and 50 PCA variables were used for either UMAP, to preserve global distances to better visualise changes across developmental stages’.

The correct version adds ‘top’ after ‘the top 2000 highly variable genes used for Principal Component Analysis (PCA), and’. This has been corrected in both the PDF and HTML versions of the Article.

The original version of this Article contained an error in ‘Data analysis’, which incorrectly read: ‘For analysis of hypothalamic patterning and generation of the developmental database HyDD (Hypothalamus Developmental Database), the E11, E12, and E13 datasets were used to perform a detailed analysis of hypothalamic patterning, and processed as described above using the top 2000 highly variable genes with 30 PCA variables’.

The correct version adds ‘top’ after ‘and processed as described above using top 2000 highly variable genes with’. This has been corrected in both the PDF and HTML versions of the Article.

The original version of this Article omitted references to previous work in ‘Methods’. This has been added as references:

[57, 63-64] at Methods, Mice: ‘*Nkx2-1*^*CreERT2*^ knock-in^57^ (JAX #014552), *Foxd1*^*CreGFP*^ knock-in^63^ (JAX #012463), *Ctnnb1*^*ex3/ex3* 64^ were used for single-cell phenotyping studies’. This has been corrected in the PDF and HTML versions of the Article.

[65] at Methods, Dissection and cell dissociation: ‘Embryos or postnatal mice were collected and dissociated using a previously published protocol^65^’. This has been corrected in the PDF and HTML versions of the Article.

[2] at Methods, Dissection and cell dissociation: ‘Prethalamus was excluded from samples aged E18 and older, with only hypothalamus collected, as previously described^2^’. This has been corrected in the PDF and HTML versions of the Article.

[65] at Methods, Dissection and cell dissociation: ‘Following dissection, tissues were dissociated in papain (Worthington Biochemical) as previously described in calcium-free Hibernate media^65’^. This has been corrected in the PDF and HTML versions of the Article.

[66-70] at Methods, Data analysis: ‘For analysis of the entire hypothalamic dataset, Seurat v3.10^66–67^ and Scanpy v1.51^68^ were used to process matrix files to keep all cells with at least 200 detected genes and 500 UMI. Datasets were normalised using Seurat ‘scTransform’ function, and Harmony v1.0^69^ was used when treating individual scRNA-Seq (orig.ident) as a variance group, to reduce inter-batch effect and adjust for individual variation.

For the entire hypothalamic dataset, the top 2000 highly variable genes were used for Principal Component Analysis (PCA), and top 50 PCA variables were used for either UMAP, to preserve global distances to better visualise changes across developmental stages^70’^. This has been corrected in the PDF and HTML versions of the Article.

[22] at Methods, Data analysis: ‘The spatial location of each neuronal cluster was identified based on transcription factor positional codes identified from HyDD analysis in combination with Allen Brain in situ atlas data analyzed using co-coframer^22^’. This has been corrected in the PDF and HTML versions of the Article.

[11, 14, 36, 71-72] at Methods, Data analysis: ‘To identify region-specific differences between mature oligodendrocytes or astrocytes of the hypothalamus and other brain regions, previously published cortical scRNA-Seq^36,71^ were used to identify differential gene expression, using age as the variance with default parameters (first-pass gene lists)^37^. Published hypothalamic datasets^11,14,36,72^ were then used to compare to these cortical scRNA-Seq datasets using the identified genes (second-pass gene lists). Identified differential genes were then validated by matching the Allen Brain in situ atlas data using co-coframer^22^ to validate spatial expression of these differential genes, as some observed differences could reflect batch effects resulting from scRNA-Seq library preparations from different laboratories (third-pass gene lists)’. This has been corrected in the PDF and HTML versions of the Article.

[14, 73-74] at Methods, Data analysis: ‘To identify the developmental origins of ependymal cells and subtypes of tanycytes, transcription factors that are highly expressed near the midpoint of the pseudotime branch - where cells are not full mature tanycytes but no longer gliogenic progenitors - were extracted and clustered to scRNA-Seq data obtained from adult tanycytes^14,73^ using Garnett v0.2.9^74^’. This has been corrected in the PDF and HTML versions of the Article.

[2, 75] at Methods, Data analysis: ‘For analysis of hypothalamic patterning and generation of the developmental database HyDD (Hypothalamus Developmental Database), the E11, E12, and E13 datasets were used to perform a detailed analysis of hypothalamic patterning^2^, and processed as described above using the top 2000 highly variable genes with top 30 PCA variables. Initial clustering was conducted using the Louvain clustering algorithm in Scanpy with 0.6 resolution^75^’. This has been corrected in the PDF and HTML versions of the Article.

[2, 21-22] at Methods, Data analysis: ‘Cross-referencing between these two pipelines allowed us to identify all the sub-regions of the developing hypothalamus, prethalamus, and adjacent structures. Following sub-divisions, enriched genes (top 50 highly-enriched genes in an individual cluster) in the individual cluster were extracted and cross-referenced back to our previous work^2^, in situ hybridization (ISH) validation, Allen Brain in situ atlas^21,22^ and GenePaint to further validate our cluster assignments^21,22^, as well as identifying pattern-specific markers’. This has been corrected in the PDF and HTML versions of the Article.

[17] at Methods, Data analysis: ‘To identify developmental hypothalamic clusters expressing neuropeptide markers, a list of neuropeptides from ^17^’. This has been corrected in the PDF and HTML versions of the Article.

[76] at Methods, Data analysis: ‘For clustering previously published datasets^76^, data was processed as previously described in the original paper’. This has been corrected in the PDF and HTML versions of the Article.

[74] at Methods, Data analysis: ‘To identify VMH cells across the entire course of hypothalamic development using the molecular stepping stone approach, gene sets identified as labeling the VMH from the E11-E13 datasets used to generate HyDD (markers that are significantly expressed in the VMH but not in ARC, Supplementary Data 4), were used to train the next developmental age (E14) and identify VMH from E14 scRNA-Seq dataset using Garnett^74^’. This has been corrected in the PDF and HTML versions of the Article.

[14, 22, 55, 73] at Methods, Data analysis: ‘This molecular stepping stone approach was validated by identification of glutamatergic VMH neurons using the public droplet-based scRNA-Seq dataset^14,73^. Spatial information of the identified clusters were validated by comparison to the Allen *Brain* in situ atlas data using co-coframer^22^, as well as by matching to the published SMART-Seq data obtained from the adult VMH.^55^ To identify the developmental origins of GABAergic neurons surrounding the core VMH (VMH-out), clusters obtained from public SMART-Seq data capturing only VMH^55^’. This has been corrected in the PDF and HTML versions of the Article.

[53, 55] at Methods, Data analysis: ‘These gene sets of the anterior and posterior VMH were validated in both our dataset at later developmental ages and in a published dataset using the molecular stepping stone approach^53^. Additional histological validation was conducted using RNAscope (described below) and using the Allen Bran in situ atlas, anterior and posterior VMH gene sets were used to train VMH clusters. Some clusters clearly labeled either anterior or posterior domains of the embryonic VMH, and were also restricted to the corresponding region of the adult VMH^55^’. This has been corrected in the PDF and HTML versions of the Article.

[74] at Methods, Data analysis: ‘For mutant phenotyping, both control and mutants of individual lines were first merged together using the above method. Given the complex phenotypes of these mutants, we used sets of region-specific markers obtained from HyDD, we trained each individual dataset using Garnett^74^’. This has been corrected in the PDF and HTML versions of the Article.

[77] at Methods, scCoGAPS analysis: ‘in order to identify cells that represent gliogenic progenitors or immature glia, or to identify VMH at individual developmental ages, using previously described parameters^77^’. This has been corrected in the PDF and HTML versions of the Article.

[78-79] at Methods, E12 spatial mapping: ‘2D spatial representation was produced using the Python-based SpatialDE package v1.1.3^78,79^’. This has been corrected in the PDF and HTML versions of the Article.

[76] at Methods, Cell cycle analysis: ‘Cell cycle data for NPCs from *Foxd1*^*Cre/+;*^*Ctnnb1*^*Ex3/+*^ mice overexpressing constitutively active *Ctnnb1* in hypothalamus and prethalamus, as well as age-matched controls, were analyzed using scran v3.11^76^’. This has been corrected in the PDF and HTML versions of the Article.

[38, 68, 80-82] at Methods, RNA velocity: ‘RNA velocity^38^ was utilized to understand the dynamic state of the entire state of hypothalamic development, gliogenesis (oligodendrocytes, astrocytes, and ependymal and tanycyte development), during when complex patterning occurs across multiple hypothalamic regions, and to identify potential differences in developmental trajectories between control and mutant samples. Kallisto and bustools^80,81^ python wrapper kb-python was used to obtain spliced and unspliced transcripts using -lamanno with GRCm38 mouse genome. Scanpy^68^ and scVelo v0.2.1^82^ was used to process the Kallisto output with default parameters, based on UMAP coordinates obtained from Seurat’. This has been corrected in the PDF and HTML versions of the Article.

[37] at Methods, Pseudotime analysis: ‘Monocle3 v0.2.0^37^ was used to perform pseudotime analysis to identify differences in gene expression in differentiating oligodendrocytes, astrocytes, and tanycytes and ependymal cells’. This has been corrected in the PDF and HTML versions of the Article.

[46] at Methods, Regulons: ‘To identify regulons controlling gene expression in different hypothalamic regions during hypothalamus patterning, SCENIC^46^ using python implemented’. This has been corrected in the PDF and HTML versions of the Article.

[2] at Methods, In situ hybridization (ISH): ‘Chromogenic in situ hybridization was performed as previously described^2^’. This has been corrected in the PDF and HTML versions of the Article.

