# Supplementary material for: The cellular and molecular landscape of hypothalamic patterning and differentiation from embryonic to late postnatal development
Source: Nat Commun. 2020 Aug 31;11:4360. doi: 10.1038/s41467-020-18231-z (PMC7459115; doi:10.1038/s41467-020-18231-z)
Supplement: Supplementary file 1 — Supplementary Information [file 41467_2020_18231_MOESM1_ESM.pdf]

Supplementary information

The cellular and molecular landscape of hypothalamic patterning and differentiation from embryonic to late postnatal development.

Dong Won Kim, Parris Whitney Washington, Zoe Qianyi Wang, Sonia Hao Lin, Changyu Sun, Basma Taleb Ismail, Hong Wang, Lizhi Jiang, and Seth Blackshaw.

Supplementary Figures:

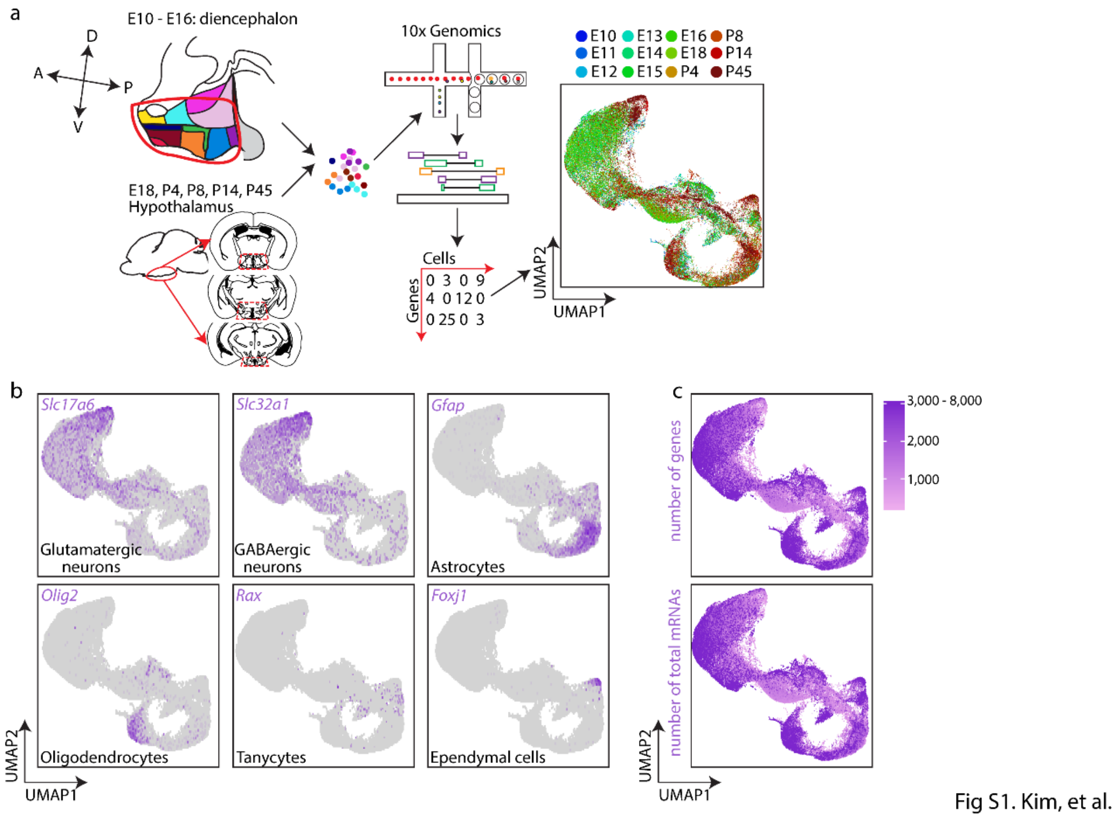

Supplementary Figure 1. Overview of a generation of the hypothalamus scRNA-Seq dataset. (a) Schematic diagram showing overall experimental strategy. 12 time points of developing diencephalon including the prethalamus and hypothalamus (between E10 and E16), and hypothalamus (between E18 and P45) were profiled using the 10x Genomics Chromium system. Distribution of individual ages (blue = younger time point, red = older time point) is shown in the UMAP plot. (b) UMAP plot showing the distribution of major cell types (purple) of the dataset. (c) UMAP plot showing the distribution of cells highlighted with a number of genes (top) or a number of cells (bottom).

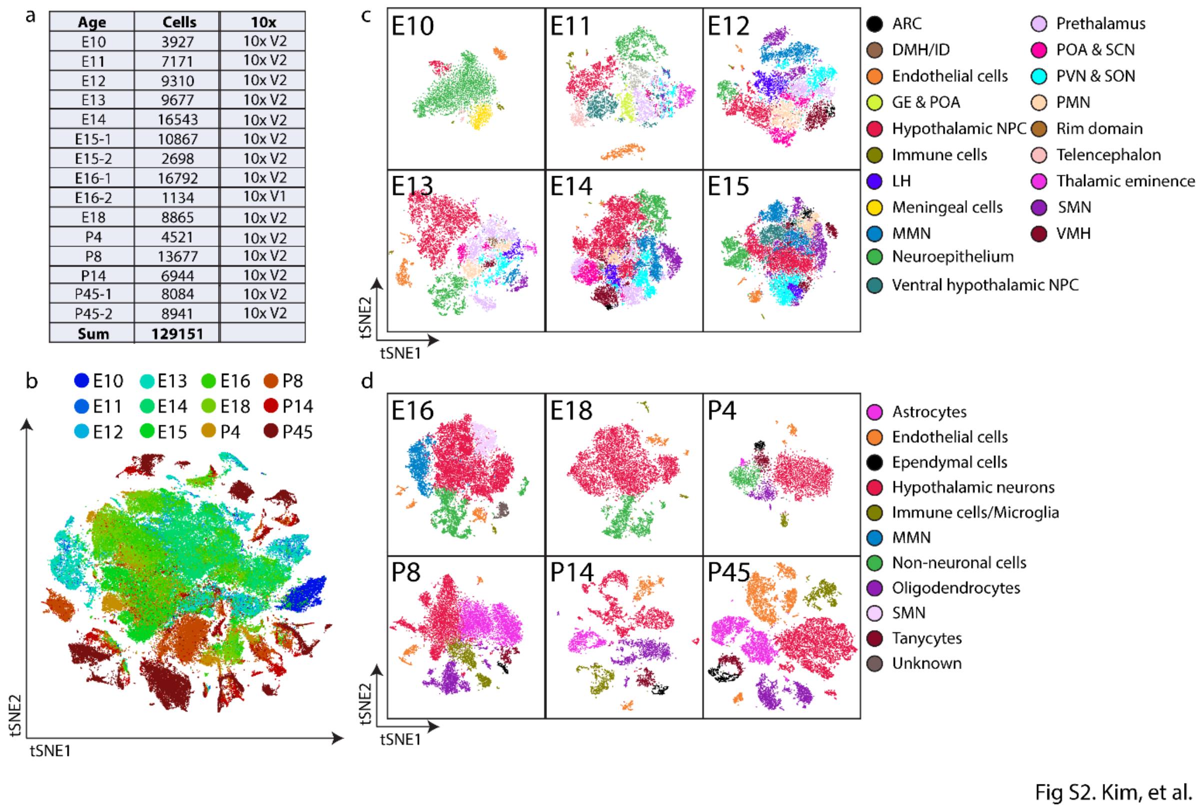

Age	Cells	10x
E10	3927	10x V2
E11	7171	10x V2
E12	9310	10x V2
E13	9677	10x V2
E14	16543	10x V2
E15-1	10867	10x V2
E15-2	2698	10x V2
E16-1	16792	10x V2
E16-2	1134	10x V1
E18	8865	10x V2
P4	4521	10x V2
P8	13677	10x V2
P14	6944	10x V2
P45-1	8084	10x V2
P45-2	8941	10x V2
**Sum**	**129151**	

Supplementary Figure 2. Overview of analysis of individual time points collected for scRNA-Seq analysis. (a) Table showing the number of cells collected for scRNA-Seq dataset. (b) tSNE showing the distribution of individual ages. (c) tSNE showing the distribution of individual prethalamic and hypothalamic regions between E10 and E15. (d) tSNE showing the distribution of individual major cell types of the hypothalamus between E16 and P45.

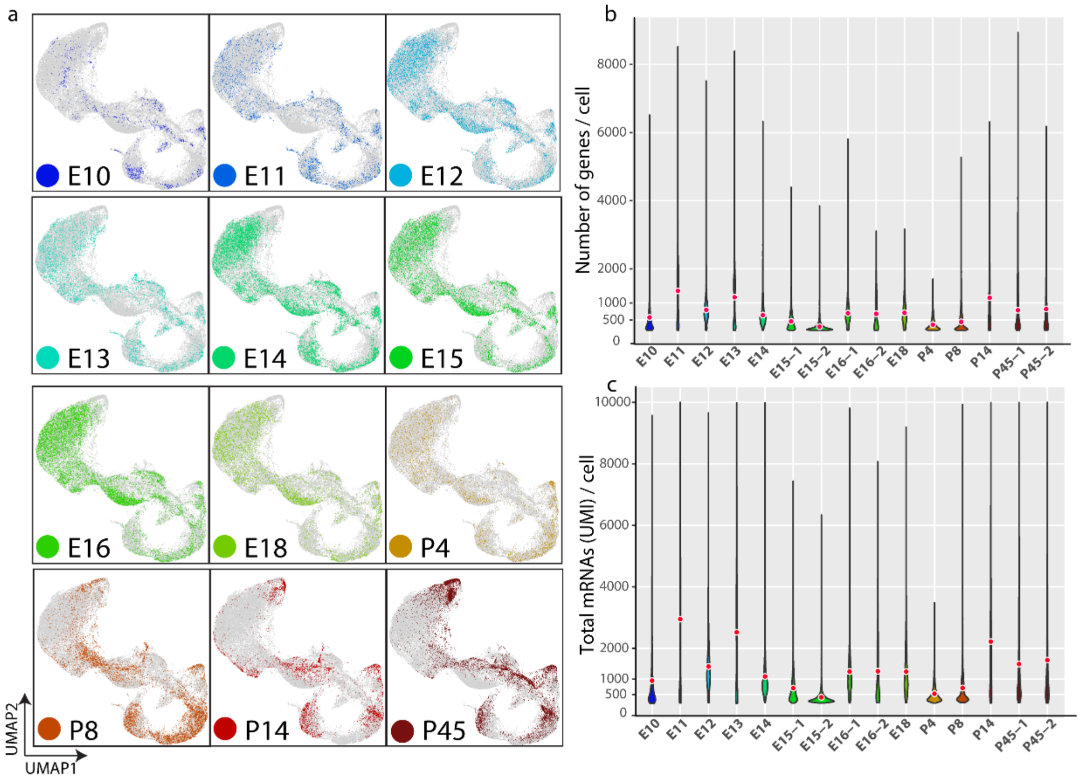

Fig S3. Kim, et al.

Supplementary Figure 3. Overview of individual time points collected for scRNA-Seq analysis. (a) UMAP plot showing the distribution of individual ages collected for scRNA-Seq. Note that the UMAP plot shows developmental trajectories. (b) Violin plot showing the distribution of mean (black dot) and of a number of genes in individual scRNA-Seq libraries. (c) Violin plot showing the distribution of mean (black dot) and a number of total mRNAs (UMI) in individual scRNA-Seq libraries.

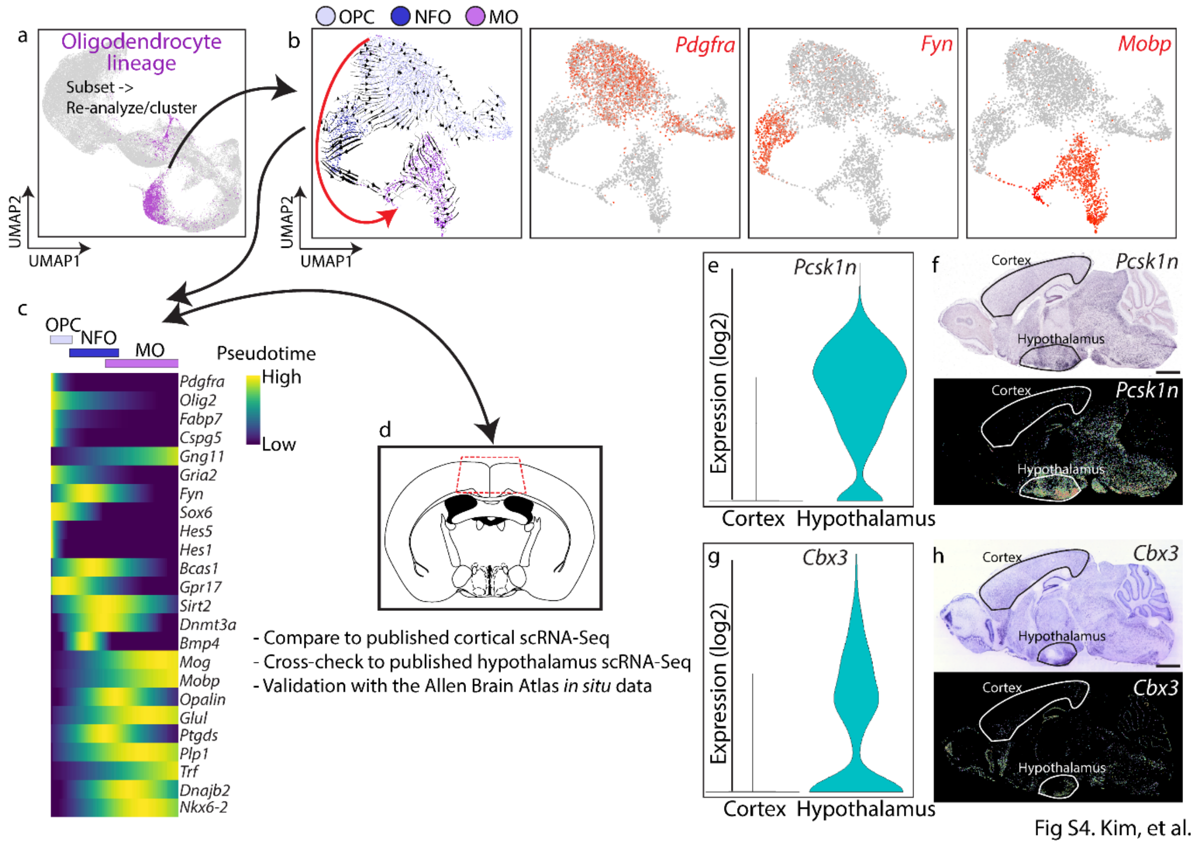

Supplementary Figure 4. Dynamics of gene expression across hypothalamus oligodendrocyte development. (a) UMAP plot showing the distribution of oligodendrocyte-lineage cells of the hypothalamus. (b) Re-clustering of hypothalamic oligodendrocyte-lineage cells showed a clear distinction of oligodendrocyte-precursor cells (OPC), newly formed oligodendrocytes (NFO) and mature oligodendrocytes (MO); with clusters being highlighted by *Pdgfra* (OPC), *Fyn* (NFO), and *Mobp* (MO) expression, and trajectories from RNA velocity analysis. (c) Pseudotime heatmap showing the dynamics of gene expression across oligodendrocyte development. (d) Hypothalamus MO cluster was compared to the MO populations identified from previously published cortical scRNA-Seq and adult hypothalamus, dataset, and cross-checked against the Allen Brain Atlas (ABA) *in situ* atlas. (e) Violin plot showing *Pcsk1n* gene expression between the cortex and hypothalamus. (f) ABA *in situ* data showing *Pcsk1n* mRNA in DIG reaction (top) and colorimetric expression (bottom). (g) Violin plot showing *Cbx3* gene expression between the cortex and hypothalamus. (h) ABA *in situ* data showing raw (top) and normalized (bottom) *Cbx3* mRNA distribution. Scale bar = 1.5 mm.

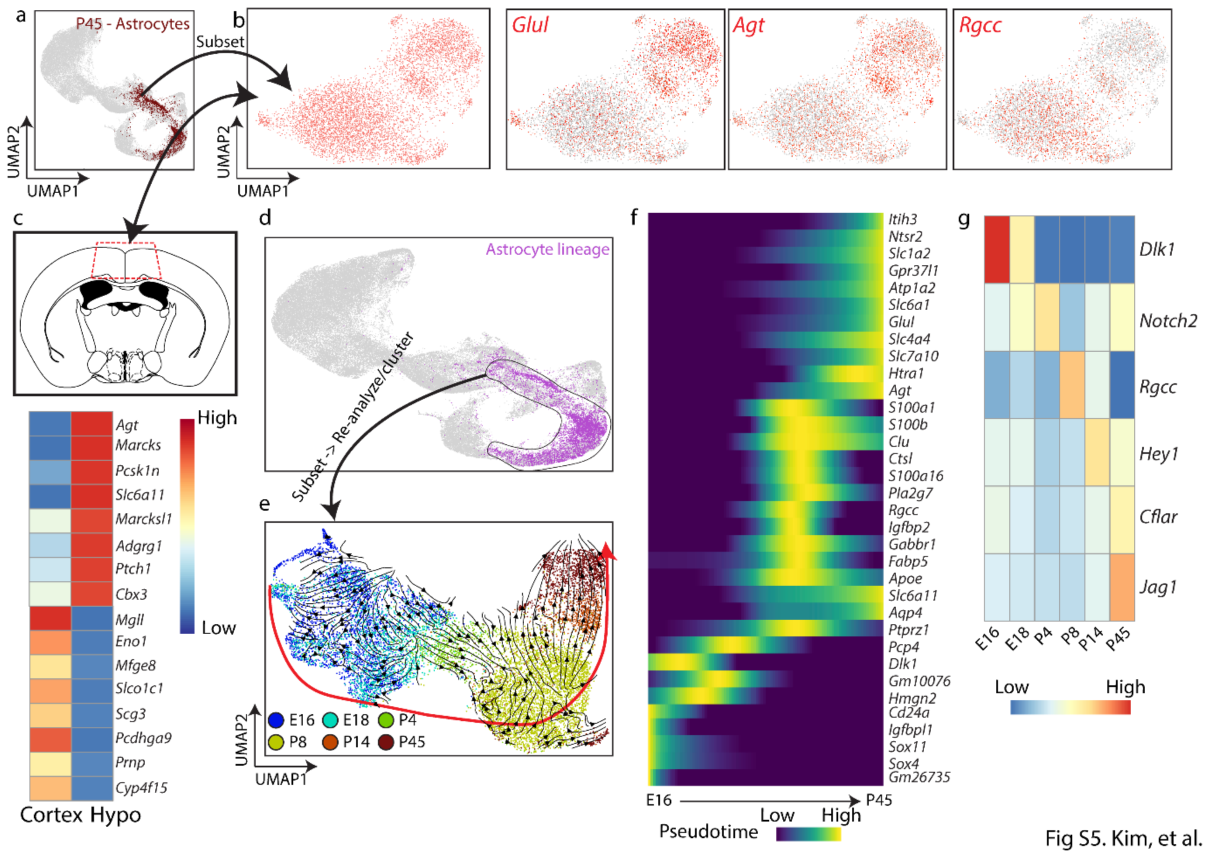

Supplementary Figure 5. Dynamics of gene expression across hypothalamus astrocyte development. (a) UMAP plot showing mature astrocytes at P45. (b) Astrocyte population was reclustered, and two distinct clusters were identified with varying expression levels of *Glul* and *Agt*, labeling more mature astrocyte clusters, and clusters with high *Rgcc* expression. (c) Hypothalamic astrocytes were compared to cortical astrocytes (top), with heatmap showing genes that are differentially expressed between these regions, a subset of which have been previously identified[1]. (d-e) The astrocyte differentiation trajectory identified in the scCoGAPS pattern highlighted by the black line was subsetted and shown in (e) with RNA velocity analysis. (f) Pseudotime heatmap showing dynamics of gene expression across hypothalamic astrocyte development. (g) Heatmap showing dynamics of Notch-related signaling genes across hypothalamic astrocyte development.

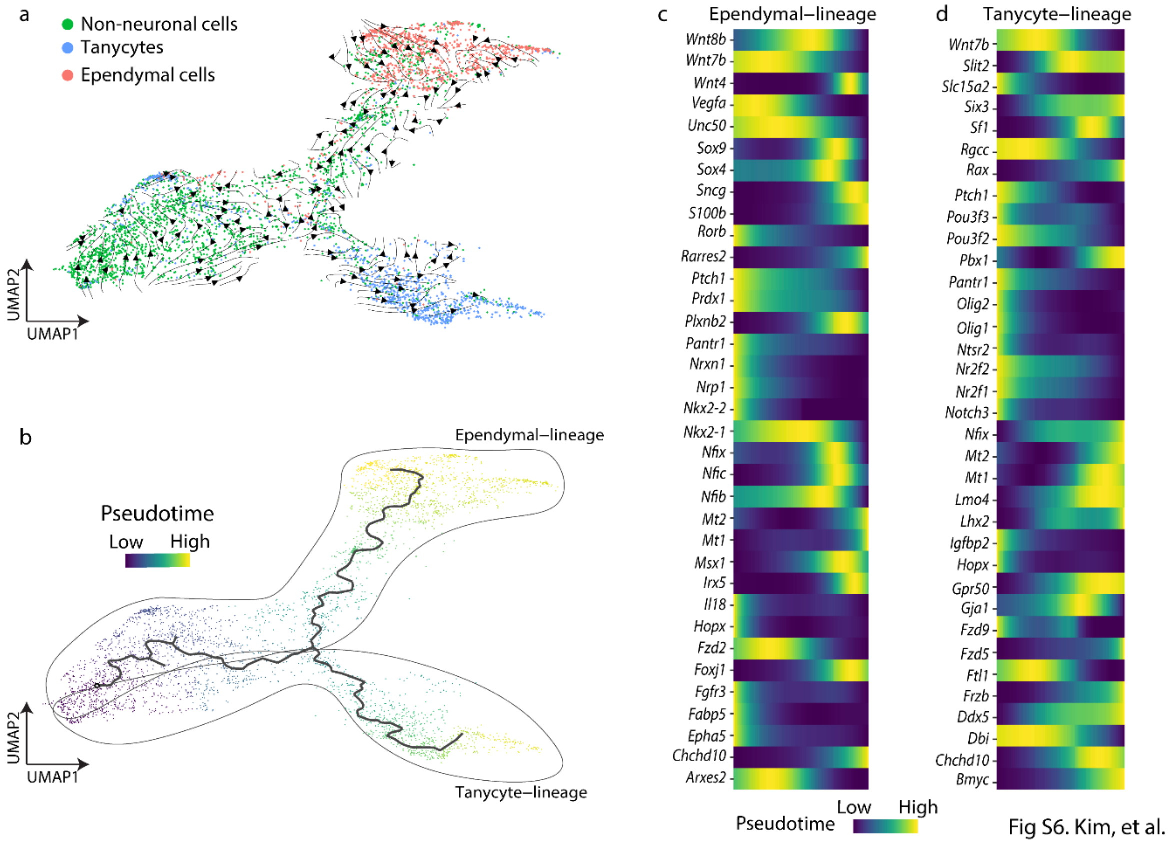

Supplementary Figure 6. Pseudotime analysis of ependymal cells and tanycytes. (a) UMAP plot with RNA velocity analysis showing trajectories of non-neuronal cells into ependymal cells or tanycytes. (b) UMAP plot with pseudotime analysis shows clear developmental trajectories of ependymal and tanycyte development. (c) Pseudotime heatmap showing gene expression changes during ependymal cell development. (d) Pseudotime heatmap showing gene expression changes during tanycyte development.

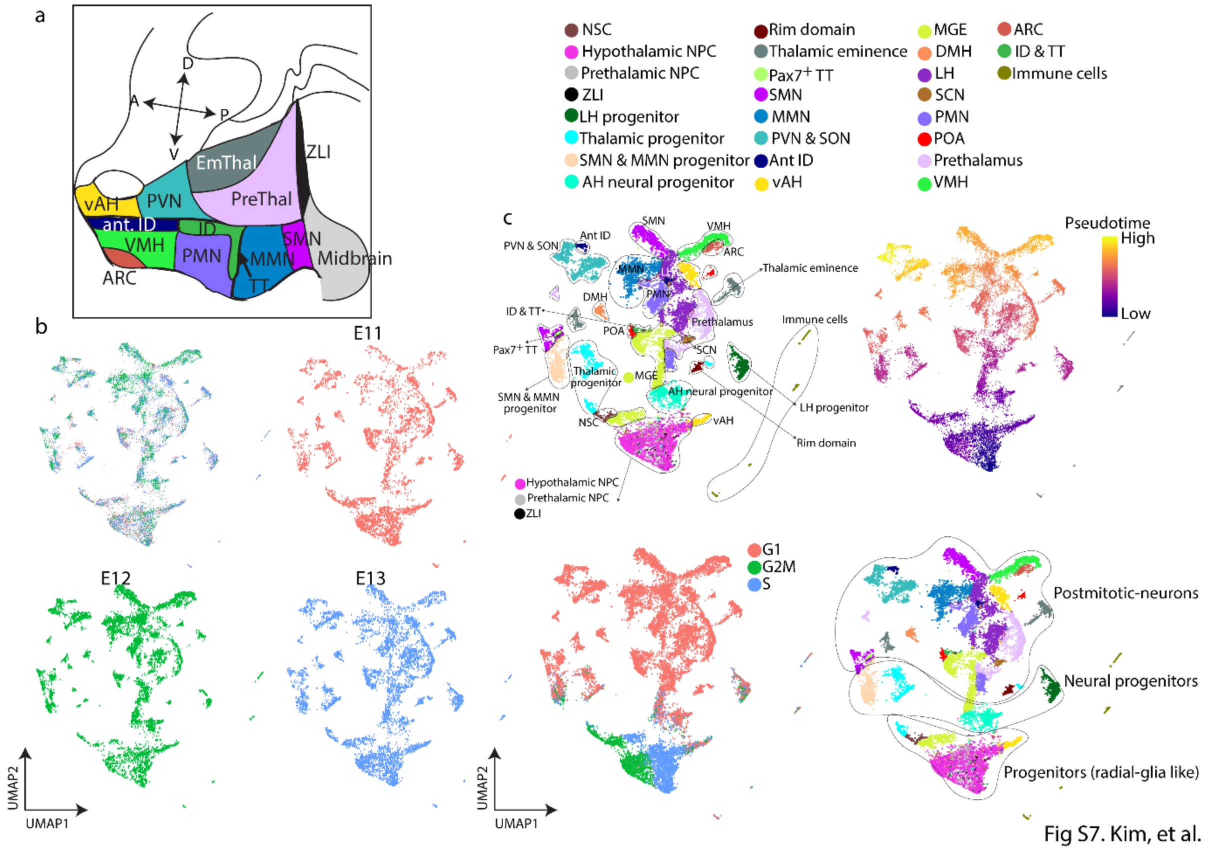

Supplementary Figure 7. Spatial divisions of the developing diencephalon. (a) Schematic sagittal plane of embryonic brain highlighting the developing diencephalon - prethalamus and hypothalamus. (b) UMAP plot highlighting cells of E11, E12, and E13. (c) UMAP plot showing the entire clusters collected at E11, E12, and E13 (top left), monocle Pseudotime plot (top right), cell cycle plot (bottom left), highlighting neural precursor cells/radial glial-like cells, neural progenitors and postmitotic neurons (bottom right).

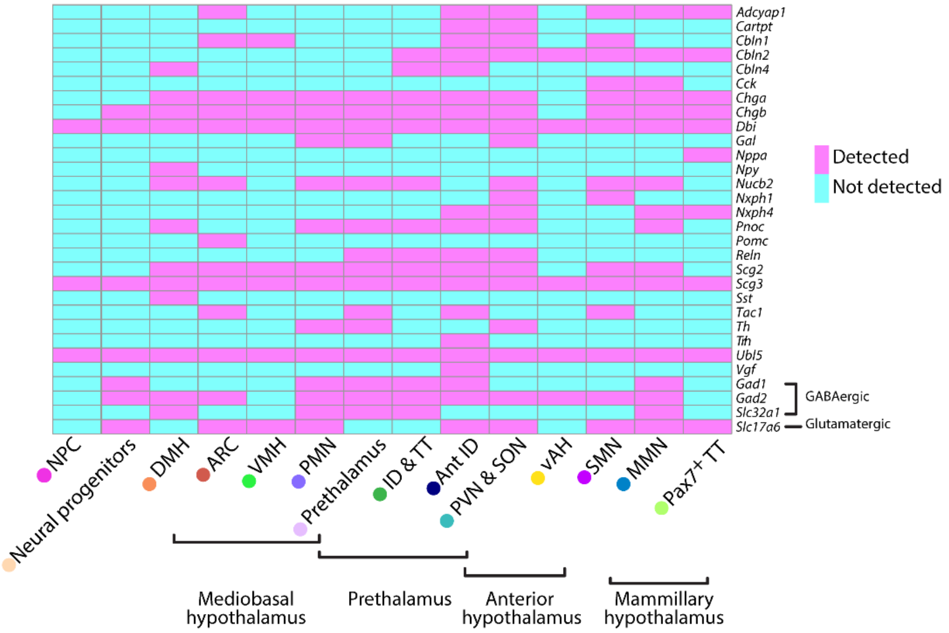

Fig S8. Kim, et al.

Supplementary Figure 8. Heatmap showing neuropeptide/neurotransmitter expression. Neuropeptide and/or neurotransmitter expression in E11-E13 1) mediobasal hypothalamus (ARC, VMH, PMN, DMH), 2) prethalamus (prethalamus, ID/TT, part of anterior ID), 3) anterior hypothalamus (part of ant ID, PVH/SON, vAH), 4) mammillary hypothalamus (SMN, MMN), are shown.

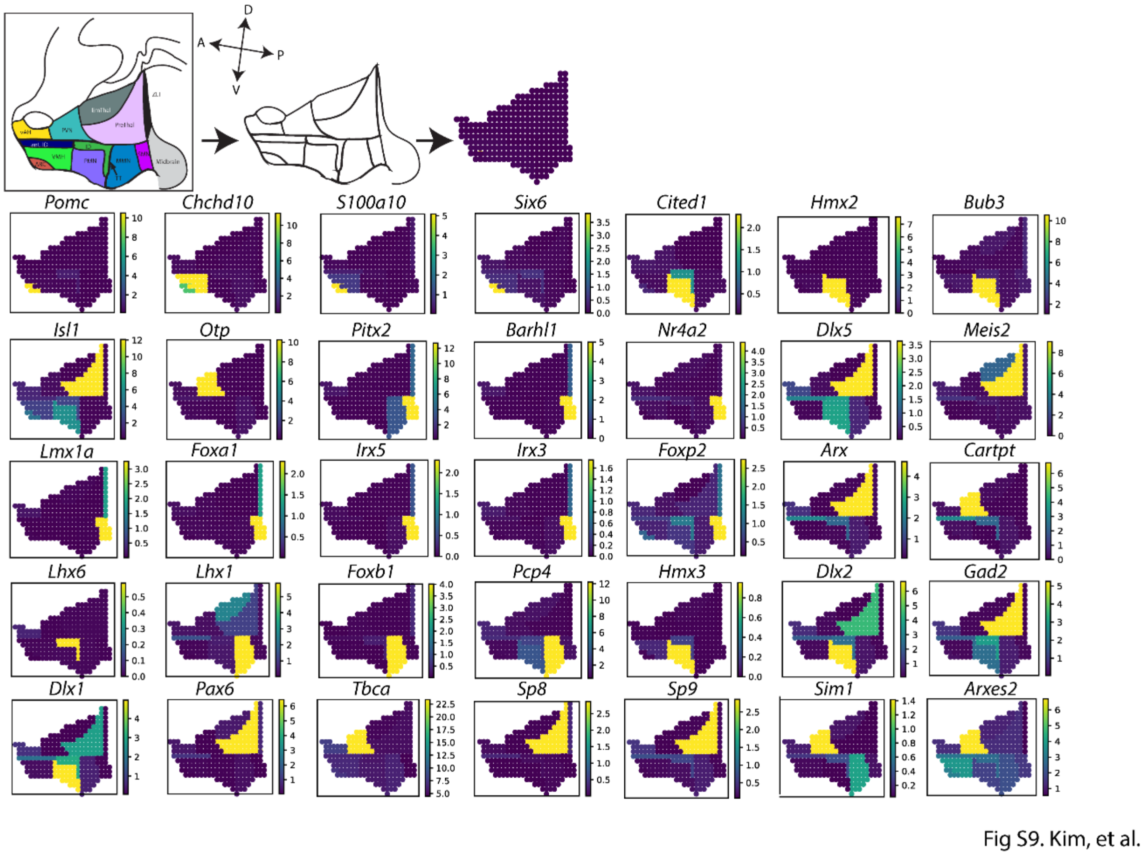

Supplementary Figure 9. 2D sagittal atlas of the developing diencephalon. Sagittal atlas of the developing hypothalamus and prethalamus, and projection of pattern-specific genes demarcating individual diencephalic regions.

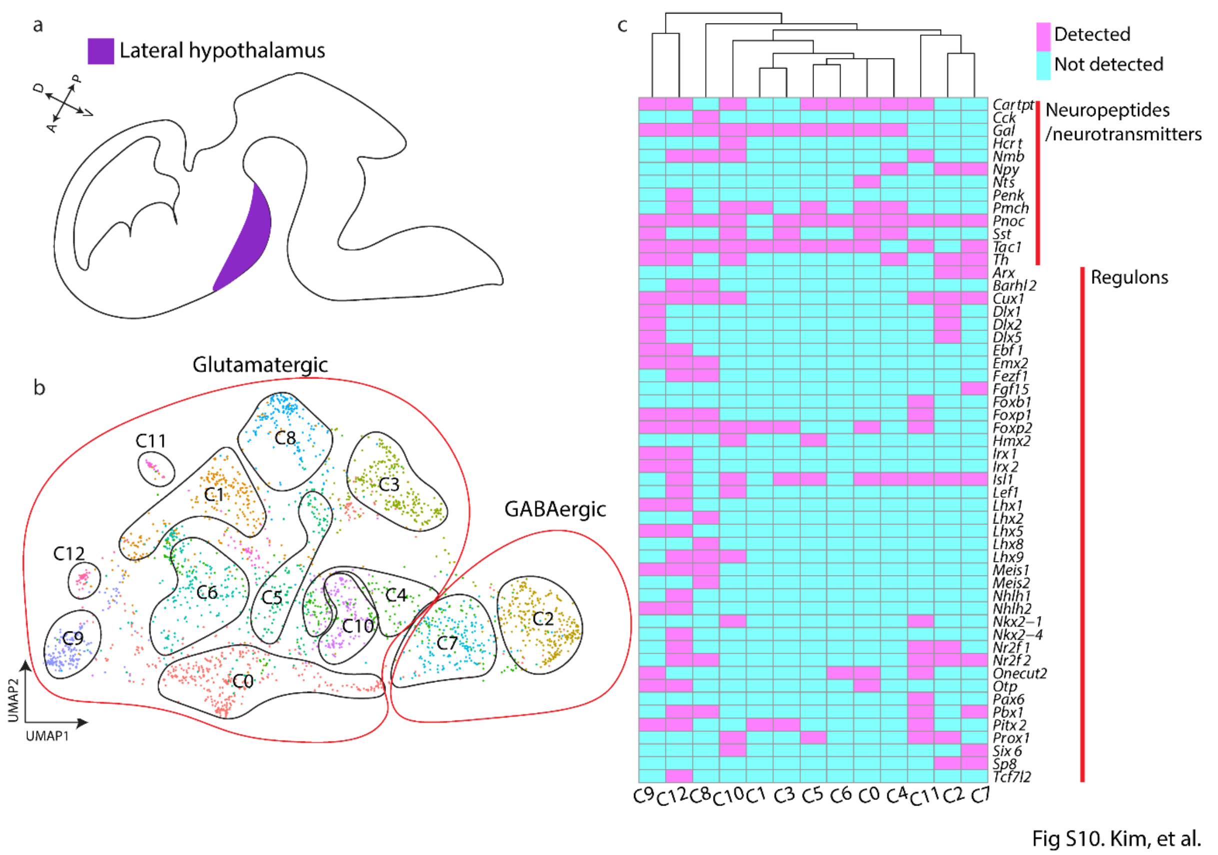

Supplementary Figure 10. Detailed analysis of LH during embryonic development (a) Schematic of mouse embryonic sagittal section highlighting the LH. (b) UMAP plot showing multiple sub-clusters of LH, comprised of either GABAergic or Glutamatergic neurons. (c) Heatmap showing neuropeptide/neurotransmitter expression detected between E11-E13 LH, as well as regulons that are key candidates of LH regulation**.**

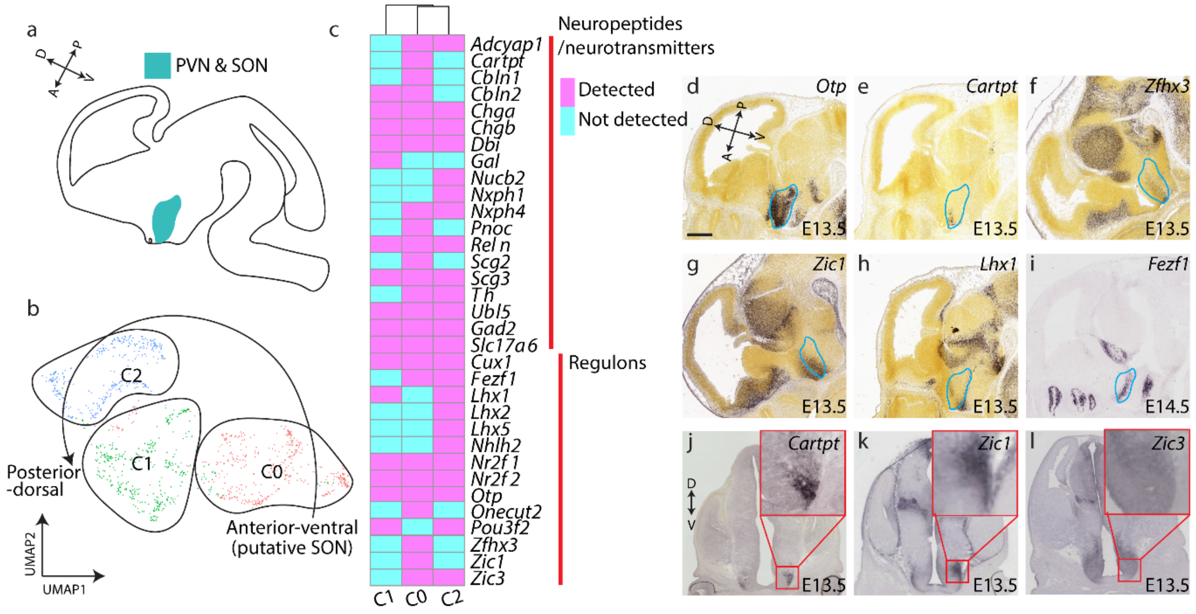

Fig S11. Kim, et al.

Supplementary Figure 11. Detailed analysis of PVN and SON during embryonic development. (a) Schematic of mouse embryonic sagittal section highlighting the PVN and SON region. (b) UMAP plot showing 3 sub-clusters of the PVN/SON. Cluster 0, located most anteriorly is putative SON. (c) Heatmap showing neuropeptide/neurotransmitter expression detected between E11-E13 PVN/SON, as well as regulons that are key candidates of PVN/SON regulation. (d-l) *In situ* hybridization from ABA (d-h), GenePaint (i) and our own data showing *Otp* (d), *Cartpt* (e), *Zfhx3* (f), *Zic1* (g), *Lhx1* (h), *Fezf1* (i), *Cartpt* – coronal plane (j), *Zic1* – coronal plane (k), *Zic3* – coronal plane (l). Scale bar = 0.4 mm.

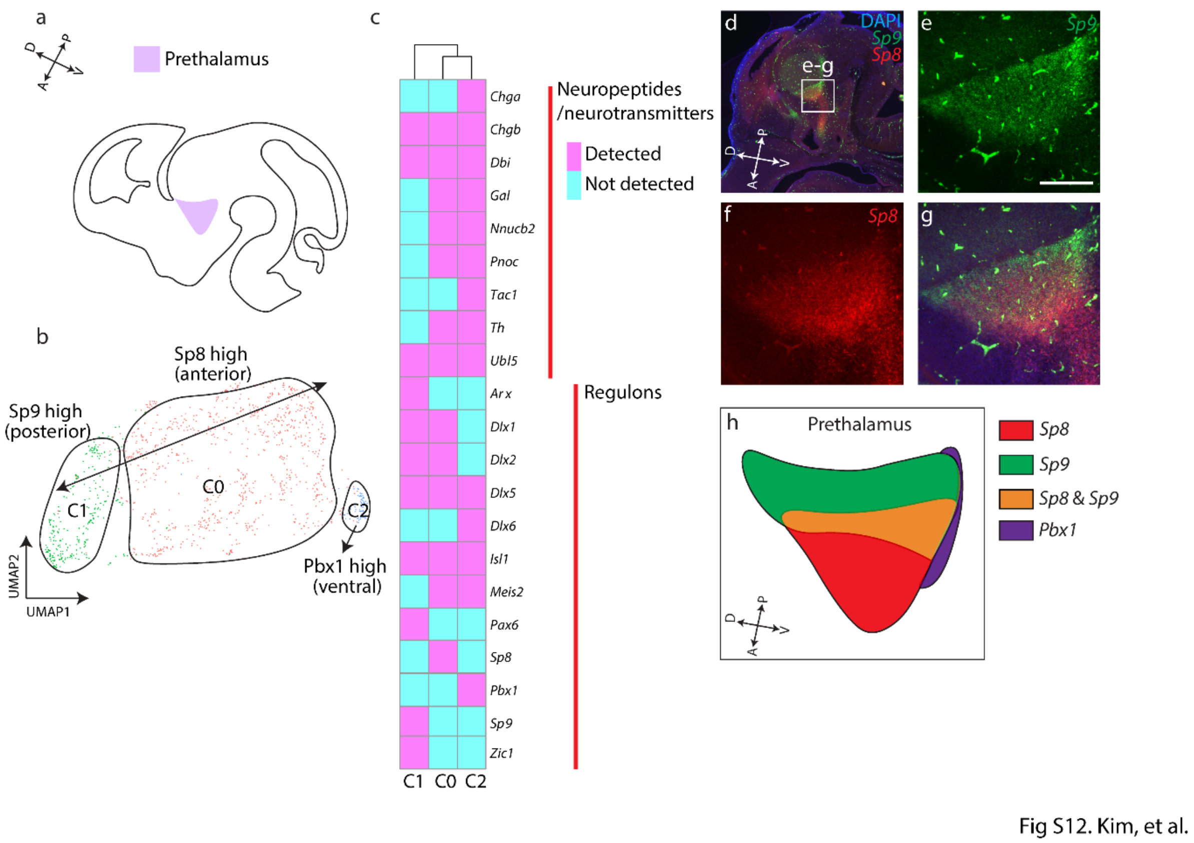

Supplementary Figure 12. Detailed analysis of prethalamus during embryonic development. (a) Schematic of mouse embryonic sagittal section highlighting the prethalamic region. (b) UMAP plot showing 3 sub-regions of the prethalamus - anterior, posterior and ventral. (c) Heatmap showing neuropeptide/neurotransmitter expression detected between E11-E13 prethalamus, as well as regulons that are key candidates of prethalamus regulation. (d-g) RNAscope shows *Sp9*-positive (e) and *Sp8*-positive (f) regions of the prethalamus. (h) Schematic of sub-regions of the developing prethalamus. Scale bar = 0.2 mm.

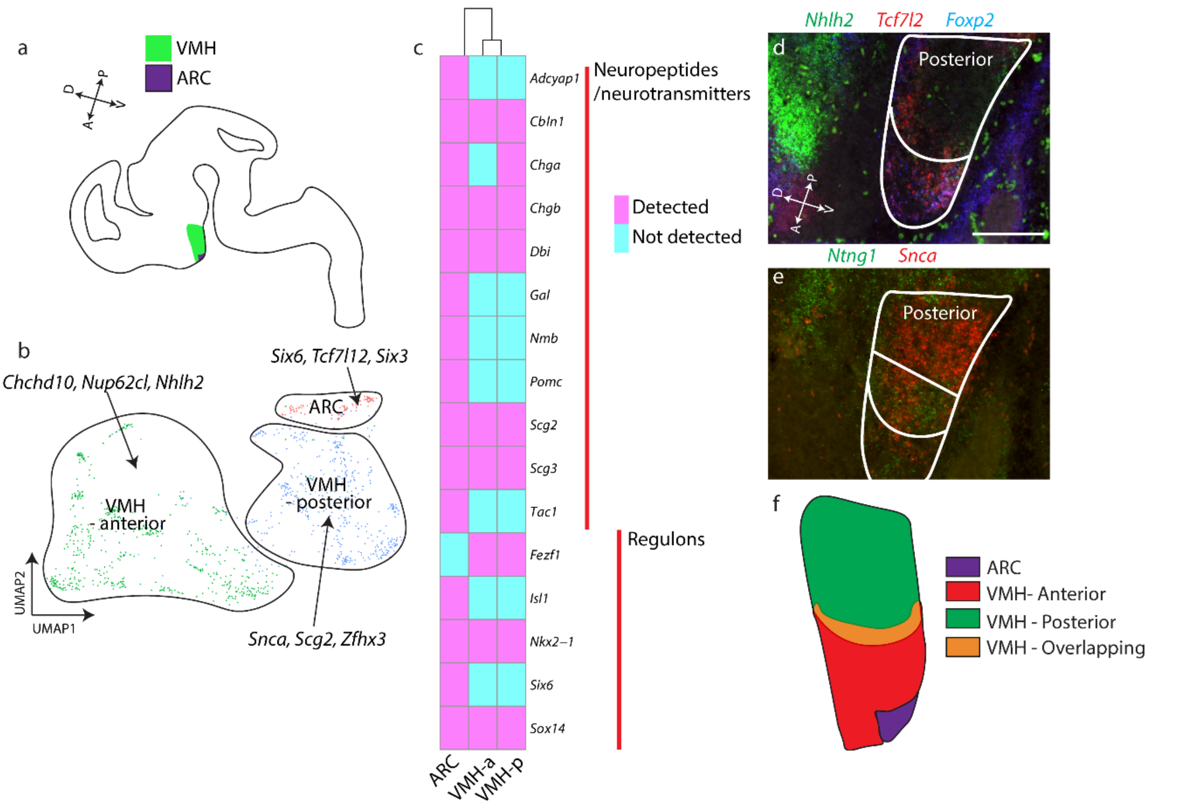

Supplementary Figure 13. Detailed analysis of VMH during embryonic development. (a) Schematic of mouse embryonic sagittal section highlighting the ARC and VMH region. (b) UMAP plot showing ARC, VMH - anterior, and VMH - posterior regions. (c) Heatmap showing neuropeptide/neurotransmitter expression detected between E11-E13 ARC and VMH, as well as regulons that are key candidates of ARC/VMH regulation. (d) RNAscope showing *Nhlh2* (green), *Tcf7l2* (red), *Foxp2* (blue), highlighting the anterior VMH region. (e) RNAscope showing *Ntng1* (green), *Snca* (red), highlighting the posterior VMH region. (f) Schematic highlighting the anterior and posterior region of the VMH. Scale bar = 0.2 mm.

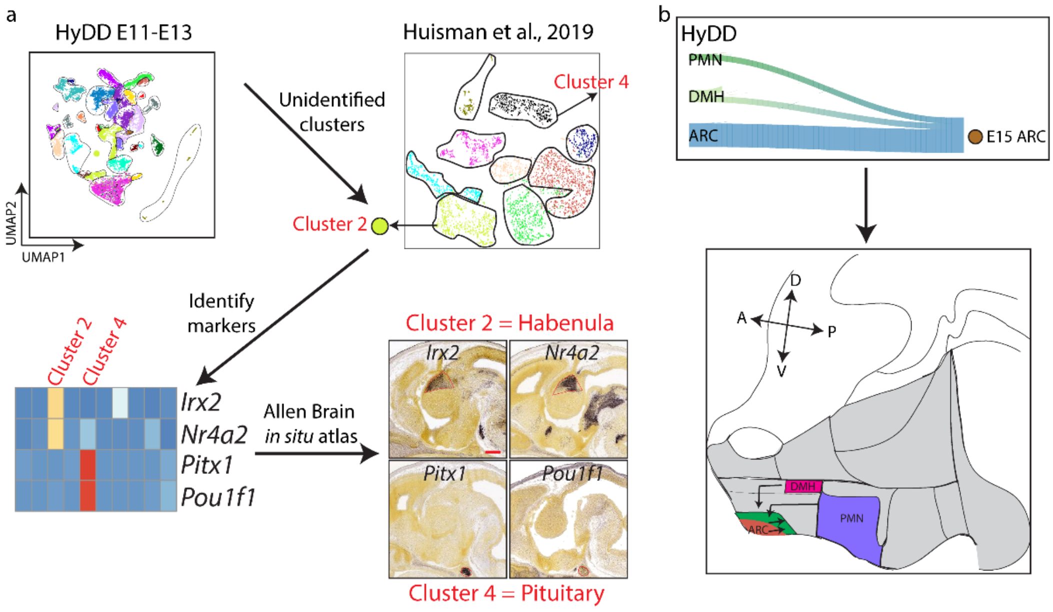

Supplementary Figure 14. Identification of un-matched clusters in public E15 hypothalamus scRNA-Seq dataset. (a) Key markers of two clusters from Huisman et al., 2019[2] that were not identified by the HyDD dataset (top) were visualized in the Allen Brain *in situ* atlas (bottom). Unmatched clusters were derived from extra-hypothalamic regions of habenula and pituitary (bottom). (b) E15 arcuate nucleus showed mixed developmental origin (top), with most populations derived from the embryonic arcuate nucleus, with some cells derived from from the PMN and DMH (bottom).

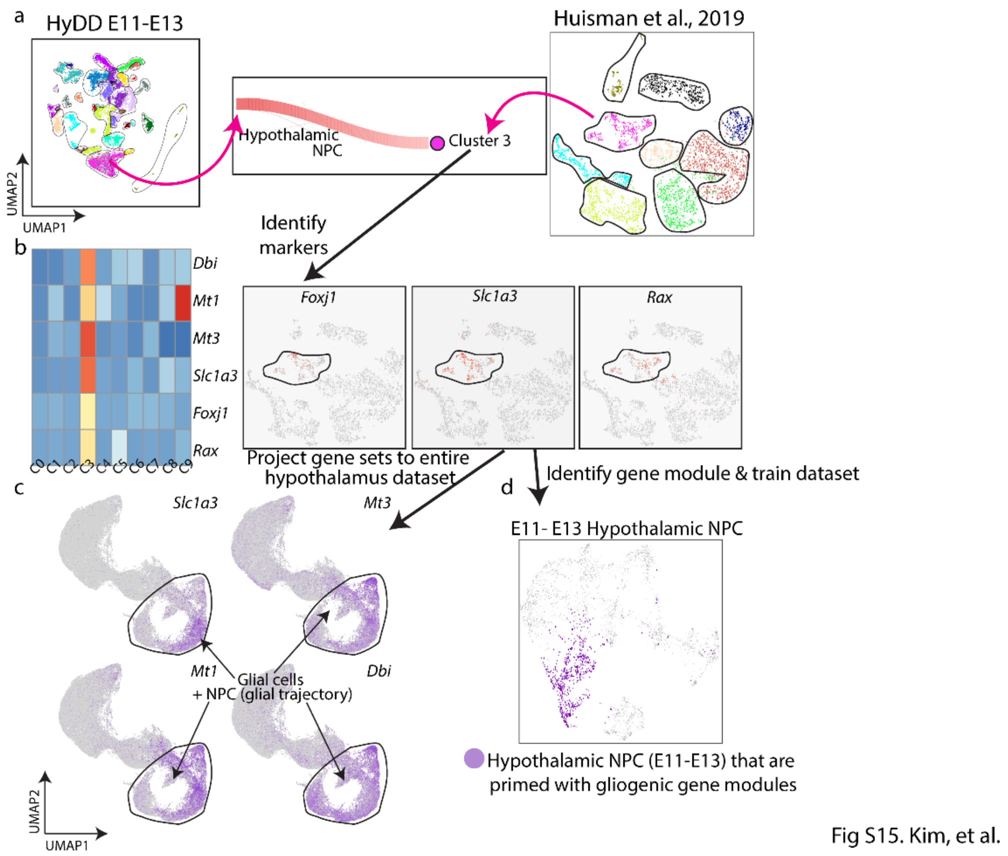

Supplementary Figure 15. Identification of hypothalamic progenitors primed with gliogenic genes. (a) Cluster 3 from Huisman et al., 2019[2] was closely related to hypothalamic NPC identified in the HyDD dataset. (b) Heatmap showing gliogenic markers of cluster 3 (left), as well as plots showing markers of ependymal cells (*Foxj1*), astrocyte/tancytes (*Slc1a3*), and tanycytes (*Rax*). (c) Visualization of key cluster 3 markers on the entire hypothalamic dataset shows the strong gene expression in glial cells and sub-populations of NPC that map to glial cell differentiation trajectories. (d) Training HyDD hypothalamic NPC with identified genes of cluster 3 shows sub-populations of NPCs that selectively express gliogenic genes.

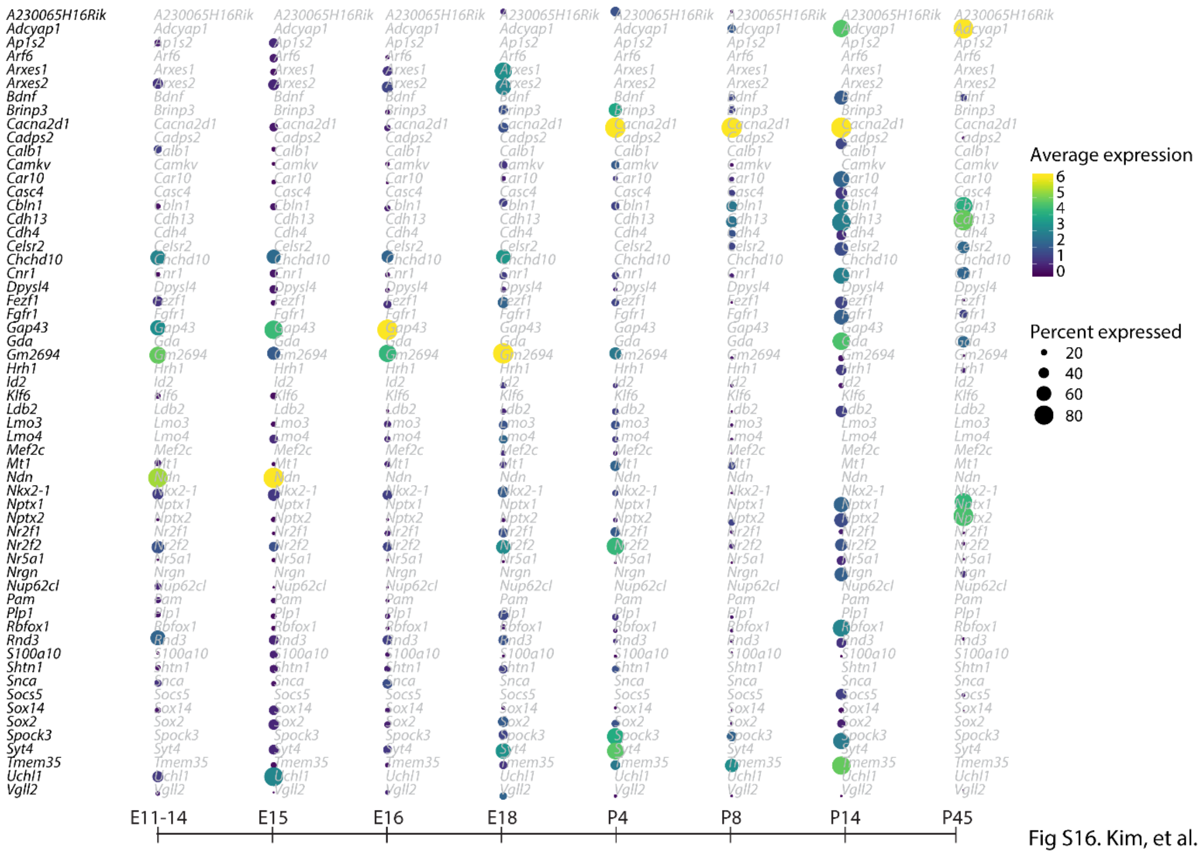

Supplementary Figure 16. Using molecular stepping stones to analyze the development of the VMH. A dot plot showing key genes that can demarcate VMH across developmental time points - some genes are expressed in multiple other regions (i.e. *Nkx2-1*), others are highly specific to the VMH (i.e. *Nr5a1*), and most genes show both varying cellular levels of expression and percentages of expression in the identified VMH across development in scRNA-Seq dataset.

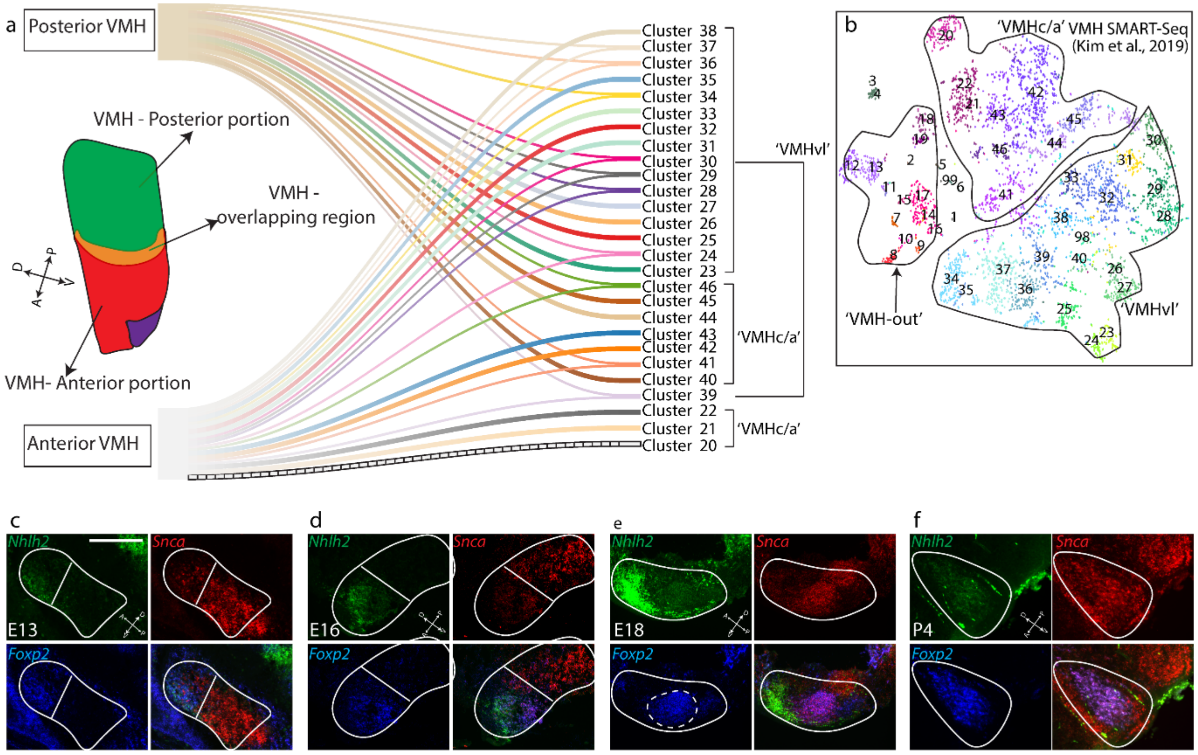

Fig S17. Kim, et al.

Supplementary Figure 17. Developmental origin of adult VMH neurons. (a) VMH clusters obtained from Kim et al., 2019[3] were trained using markers that selectively label anterior and posterior domains of the developing VMH to identify their developmental origin. (b) tSNE plot showing the distribution of VMH SMART-Seq from Kim et al., 2019[3]. VMH clusters are derived from the original paper. (c-f) RNAscope shows distinct anterior-posterior regions of the VMH at E13 (c) and E16 (d) based on the anterior VMH markers *Nhlh2* and *Foxp2* and the posterior VMH marker *Snca*. From E18 (e) onwards, anterior VMH regions start to intermingle with posterior VMH regions. At P4 (f), anterior and posterior VMH regions are extensively intermixed. Scale bar = 0.2 mm (c), 0.25 mm (d), 0.3 mm (e) 0.4 mm (f).

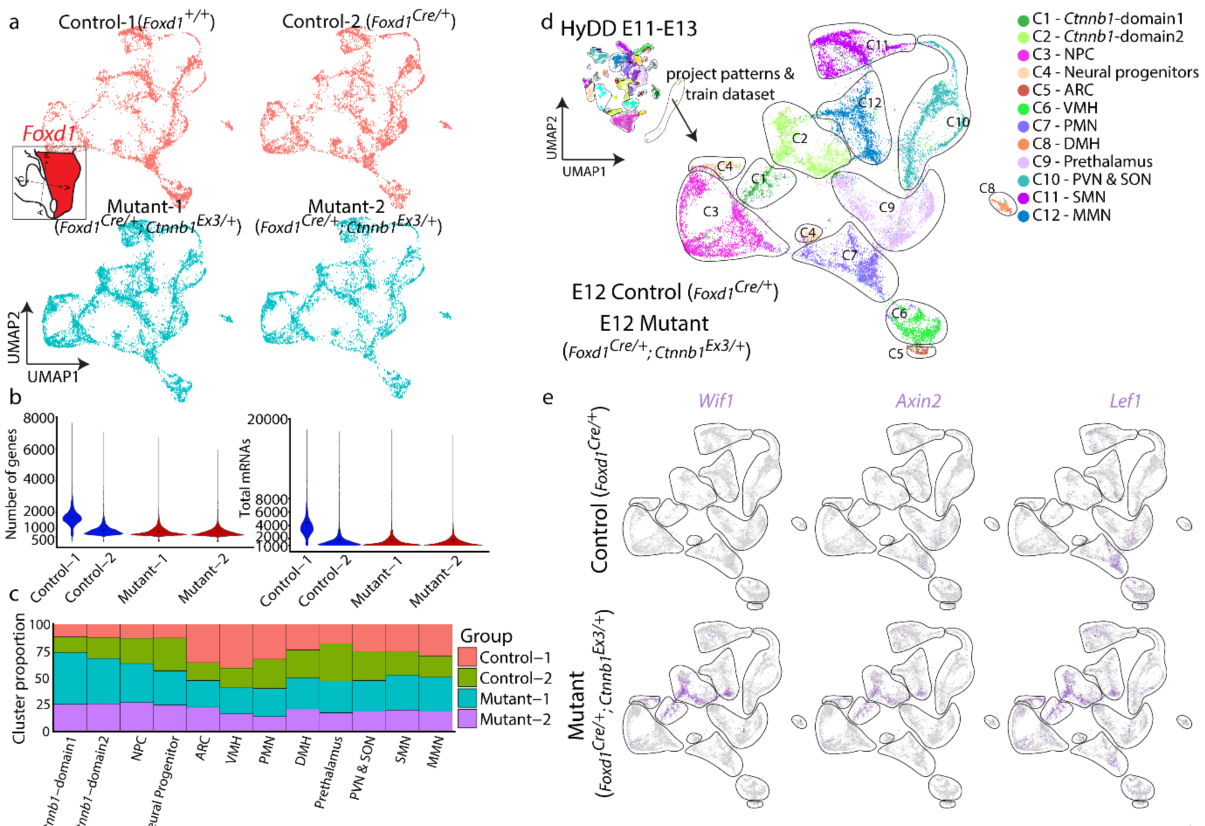

Fig S18. Kim, et al.

Supplementary Figure 18. scRNA-Seq can be used to screen mutant phenotype in the hypothalamus. (a) UMAP plot showing the distribution of cells in individual scRNA-Seq libraries in control (*Foxd1*$^{Cre/+}$) and mice selectively overexpressing a constitutively active mutant form of *Ctnnb1* in neuroepithelial cells of the hypothalamus and prethalamus (*Foxd1*$^{Cre/+}$;*Ctnnb1*$^{Ex3/+}$) . (b) Violin plot showing the distribution of mean (black dot) and a number of genes in individual scRNA-Seq libraries (left). Violin plot showing the distribution of mean (black dot) and a number of total mRNAs (UMI) in individual scRNA-Seq libraries (right). (c) Bar graph showing the distribution of individual scRNA-Seq libraries across hypothalamic clusters. (d) UMAP plot showing clusters from the combined scRNA-Seq dataset of control and mutants, in which clusters were obtained by training the dataset with HyDD markers. (e) UMAP plot showing high levels of *Wnt*-signaling genes, *Wif1*, *Axin2*, *Lef1*, in 2 clusters (*Ctnnb1*-domain 1 and 2) that are occupied by constitutively active *Ctnnb1* mutants.

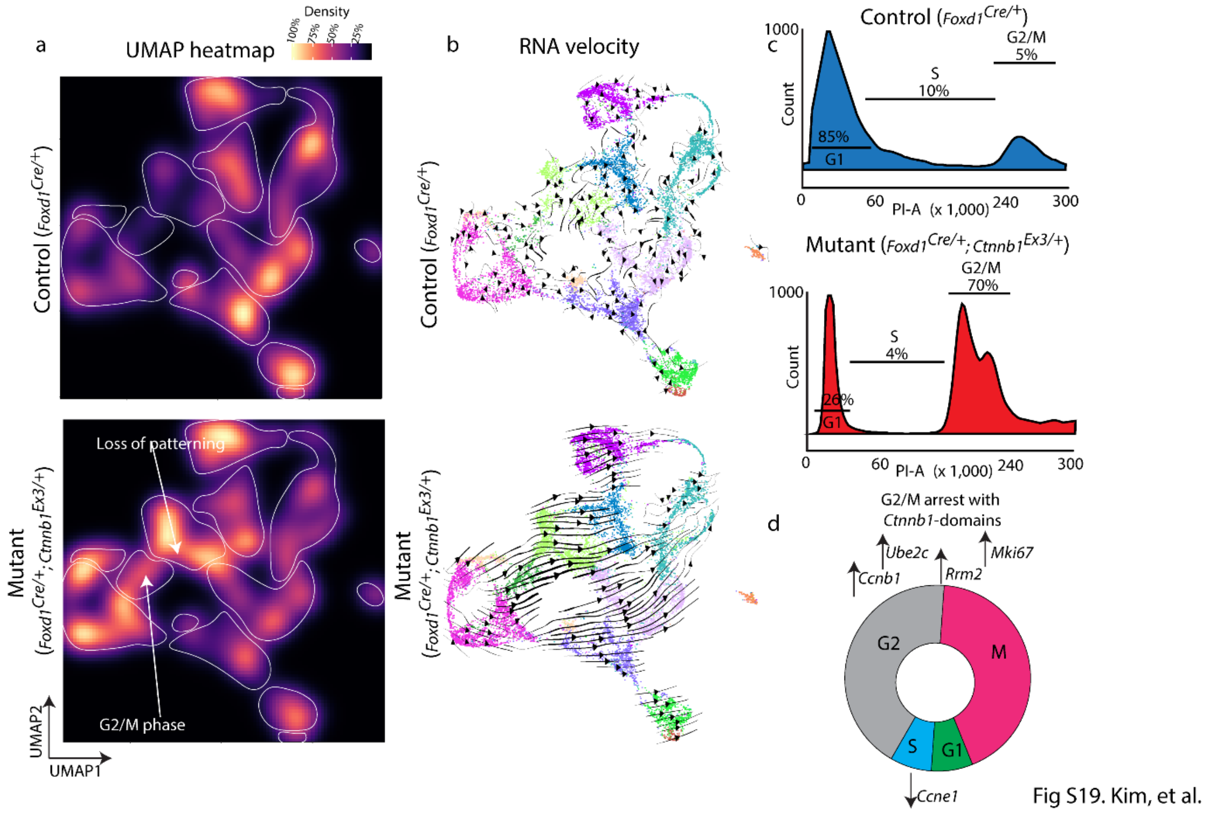

Supplementary Figure 19. Disrupted cell cycle in constitutively active *Ctnnb1* mutants. (a) UMAP heatmap plot showing distribution of individual clusters between control (*Foxd1*$^{Cre/+}$)(top) and constitutively active *Ctnnb1* mutants (*Foxd1*$^{Cre/+}$;*Ctnnb1*$^{Ex3/+}$)(bottom). (b) UMAP plot with RNA velocity analysis in control (*Foxd1*$^{Cre/+}$)(top) and constitutively active *Ctnnb1* mutants (*Foxd1*$^{Cre/+}$;*Ctnnb1*$^{Ex3/+}$)(bottom). Note trajectory from NPC to constitutively active *Ctnnb1* mutants-enriched clusters. (c) Flow cytometry data in combination with propidium iodide (PI) staining to show cells at different stages of the cell cycle in control (top) and mutant samples (bottom). (d) Constitutively active *Ctnnb1* mutants-enriched clusters show increased G2/M-phase markers and reduction of G1/S-phase markers.

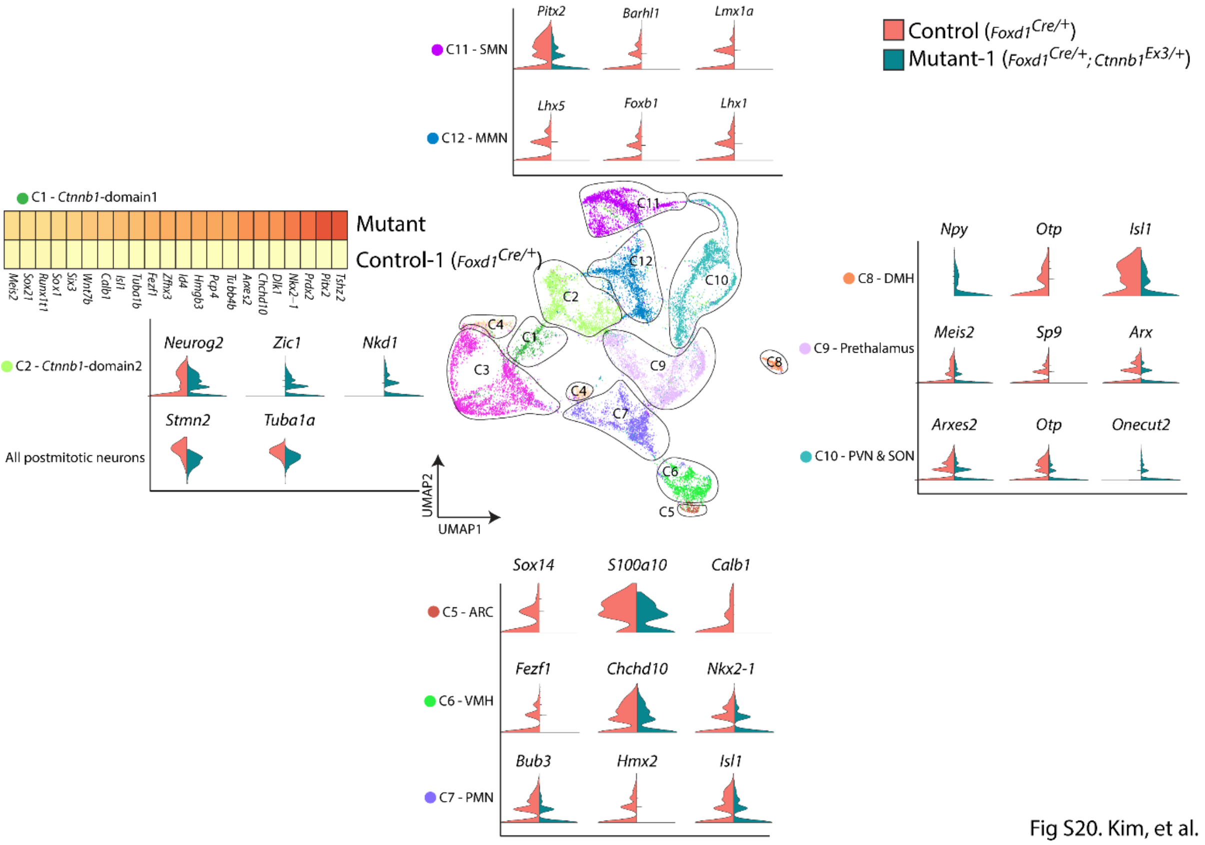

Supplementary Figure 20. Differential gene expression between control and constitutively active *Ctnnb1* mutants. Violin plots showing gene expression differences between control and constitutively active *Ctnnb1* mutants.

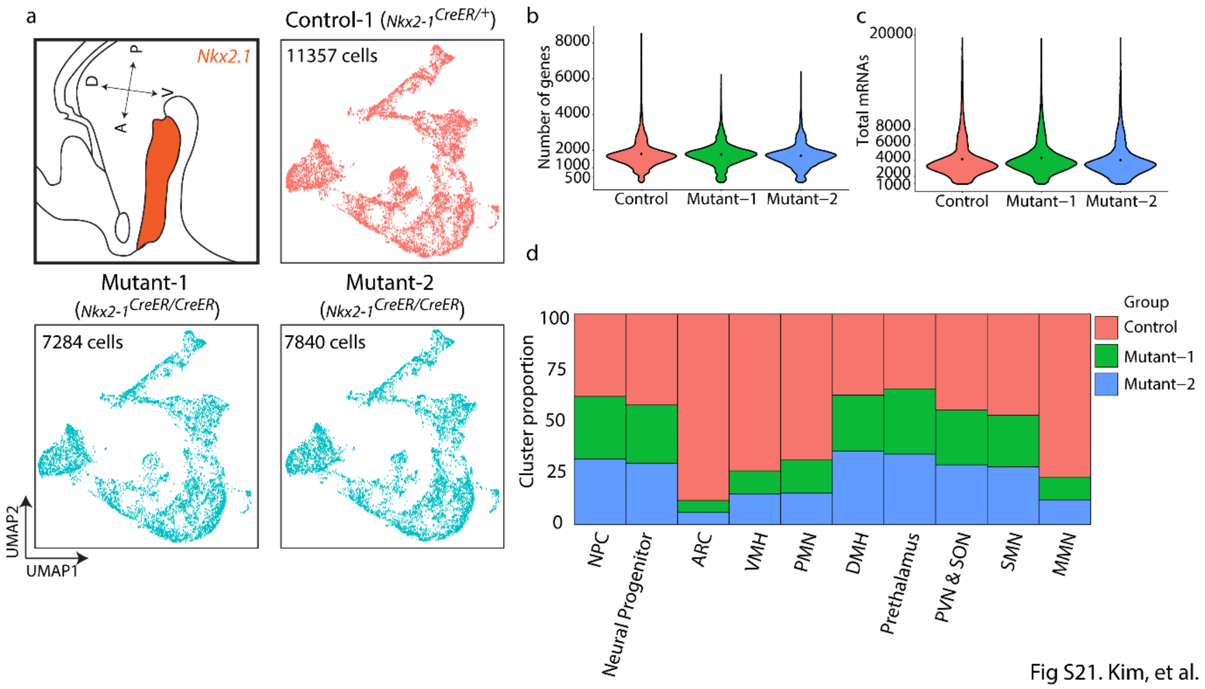

Supplementary Figure 21. Overview of individual cell clusters in *Nkx2-1* mutants. (a) UMAP plot showing the distribution of cells in individual scRNA-Seq libraries. (b) Violin plot showing the distribution of mean (black dot) and number of genes in individual scRNA-Seq libraries. (c) Violin plot showing the distribution of mean (black dot) and a number of total mRNAs (UMI) in individual scRNA-Seq libraries. (d) Bar graph showing cluster distribution between individual scRNA-Seq libraries.

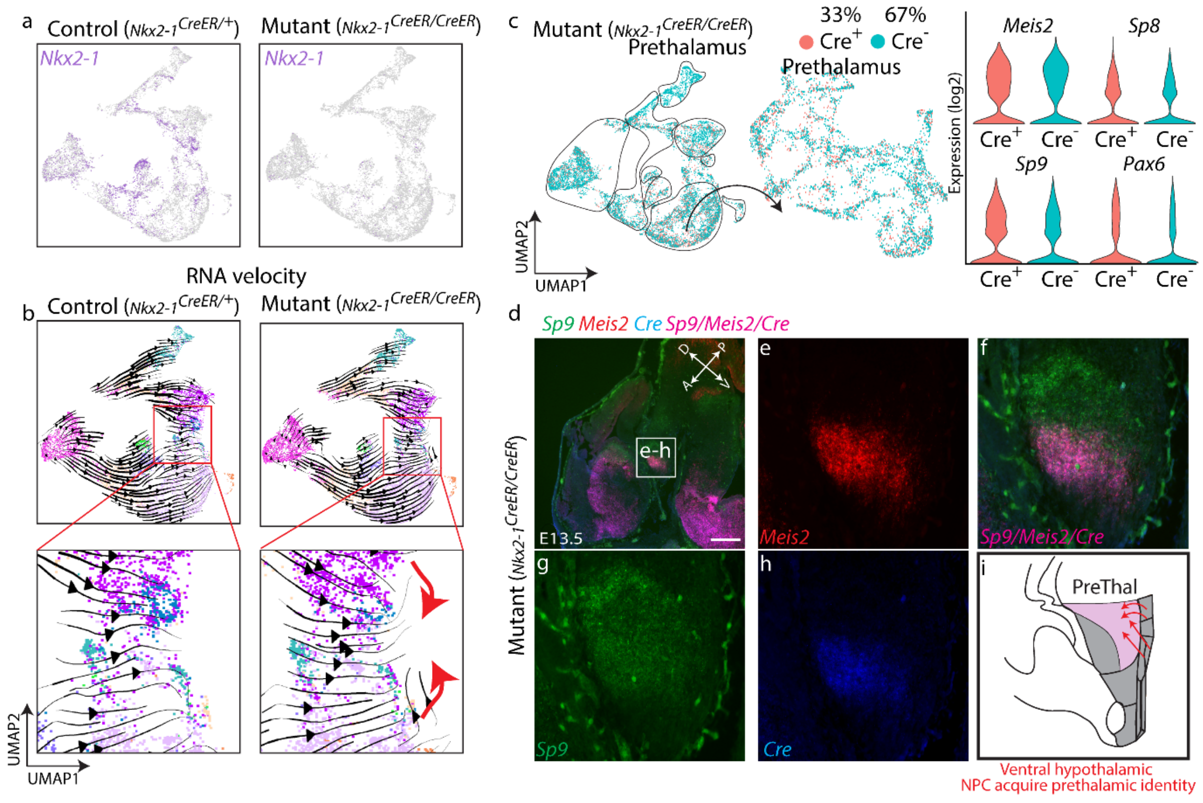

Fig S22. Kim, et al.

Supplementary Figure 22. Characterization of *Nkx2-1* mutants. (a) UMAP plot showing the absence of *Nkx2-1* expression in mutant mice. (b) UMAP plot with RNA velocity analysis of control and *Nkx2-1* mutants. Note different trajectories from progenitors to MMN and prethalamus in *Nkx2-1* mutants. (c) UMAP plot showing cells in both controls and *Nkx2-1* mutants p expressing *Cre* (left), with *Nkx2-1*-deficient prethalamic cells highlighted (middle). Violin plots show no significant differences between expression levels of prethalamic markers between Cre-positive and Cre-negative prethalamic cells in *Nkx2-1* mutants. (d-f) RNAscope showing *Cre* expression in *Sp9*+ and *Meis2*+ prethalamic cells in *Nkx2-1* mutants (d-h), and schematic (i) showing the conversion of ventral hypothalamic NPCs to prethalamic identity in the absence of *Nkx2-1*. Scale bar = 0.2 mm.

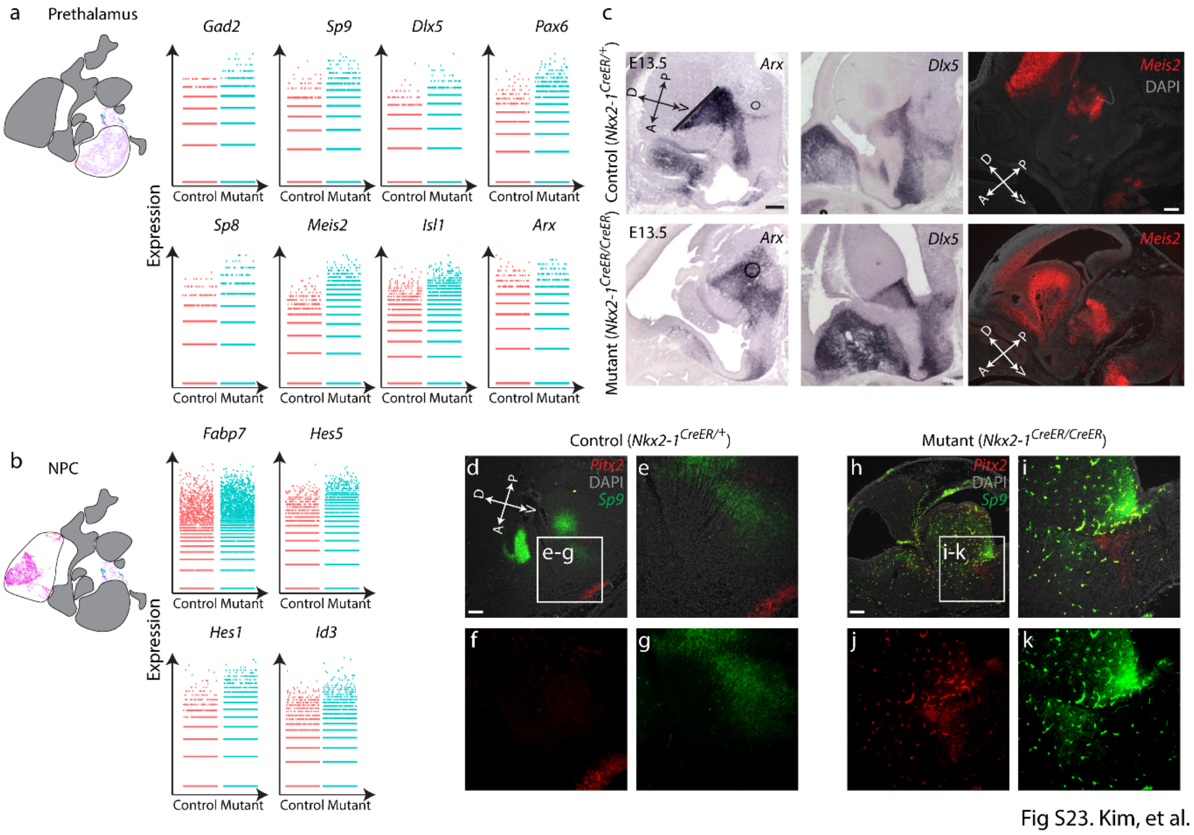

Supplementary Figure 23. *Nkx2-1* mutants show an increase in prethalamic cells. (a) UMAP plot showing the location of the prethalamus with jitter plots of the prethalamus marker genes (*Gad2*, *Sp9*, *Dlx5*, *Pax6, Sp8*, *Meis2, Isl1*, *Arx*). (b) UMAP plot showing the location of the NPC with jitter plots of the NPCmarker genes (*Fabp7*, *Hes5, Hes1*, *Id3*). (c) *In situ* hybridization showing *Arx*, *Dlx5*, and *Meis2* in control (top) and *Nkx2-1* mutants (bottom). (d-k) *In situ* hybridization showing *Pitx2* and *Sp8* in control (d-g) and *Nkx2-1* mutants (h-k). Scale bar = 0.2 mm.

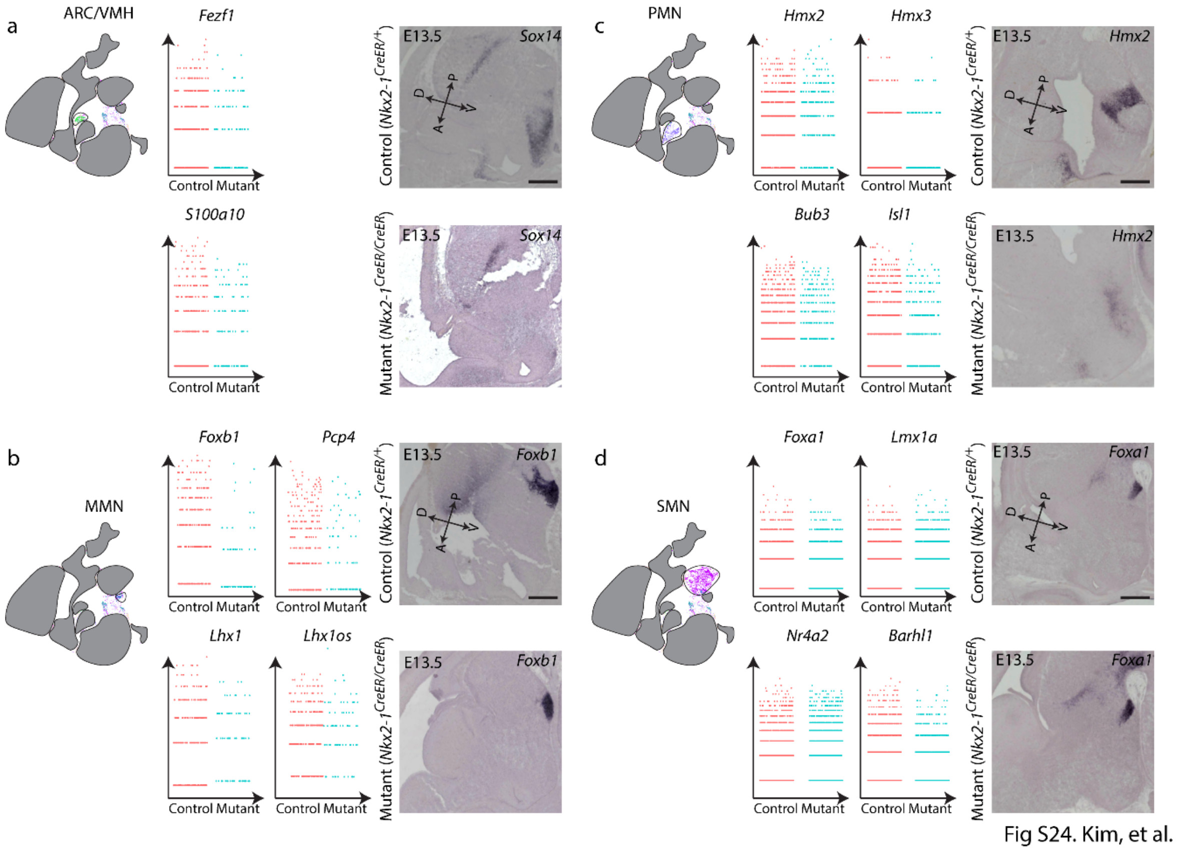

Supplementary Figure 24. *Nkx2-1* mutant shows a reduction in Nkx2.1-expressing posteroventral hypothalamic structures with the exception of the supramammillary nucleus. (a) UMAP plot showing the location of the ARC/VMH with jitter plots of the ARC/VMH marker genes (*Fezf1*, *S100a0*), and *in situ* hybridization showing *Sox14* in control (top) and *Nkx2-1* mutants (bottom). (b) UMAP plot showing the location of the MMN with jitter plots of the MMN marker genes (*Foxb1*, *Pcp4, Lhx1*, *Lhx1os*), and *in situ* hybridization showing *Foxb1* in control (top) and *Nkx2-1* mutant (bottom). (c) UMAP plot showing the location of the PMN with jitter plots of the PMN marker genes (*Hmx2*, *Hmx3, Bub3*, *Isl1*), and *in situ* hybridization showing *Hmx2* in control (top) and *Nkx2-1* mutant (bottom). (d) UMAP plot showing the location of the SMN with jitter plots of the SMNmarker genes (*Foxa1*, *Lmx1a, Nr4a2*, *Barhl1*), and *in situ* hybridization showing *Foxa1* in control (top) and *Nkx2-1* mutants (bottom). Scale bar = 0.4 mm.

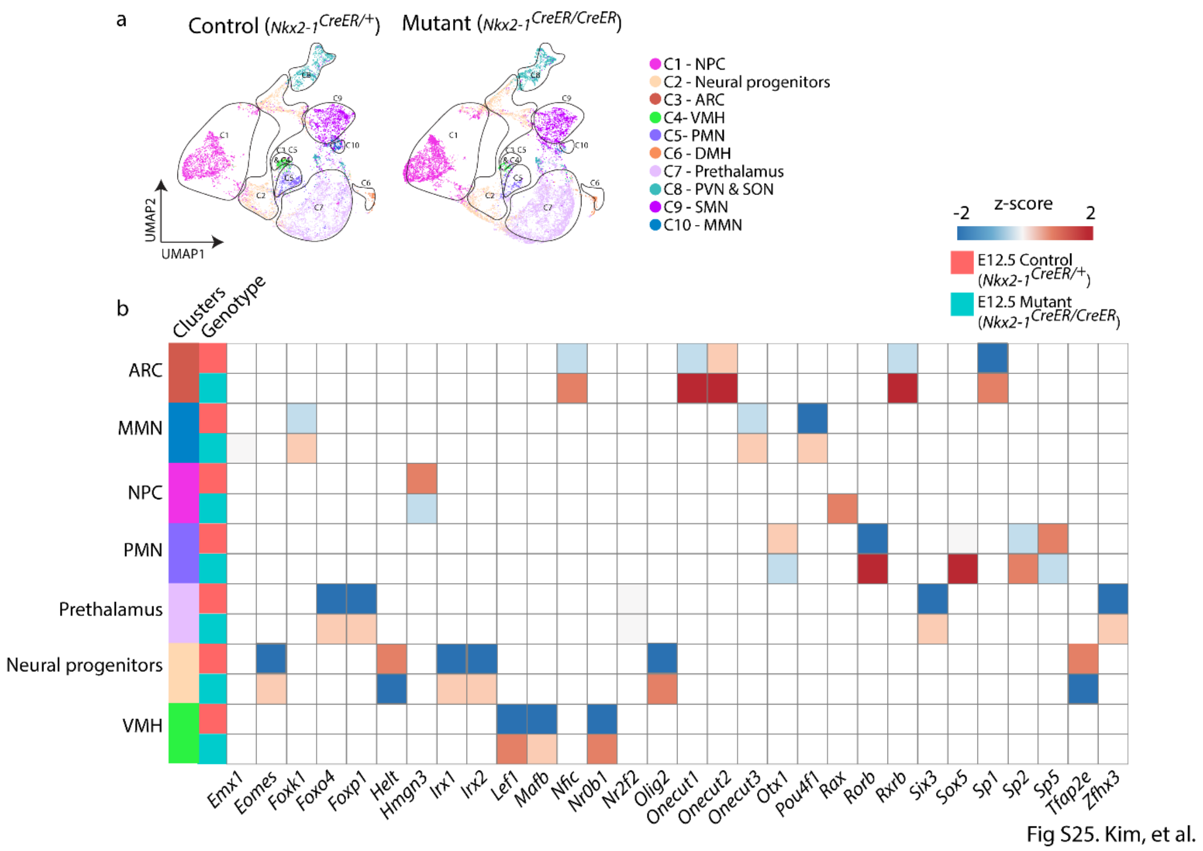

Supplementary Figure 25. *Nkx2-1* mutant line shows altered regulons. (a) UMAP plot showing clusters of control and *Nkx2-1* mutants. (b) Heatmap showing z-scores of significantly differentially expressed regulons between clusters of control and *Nkx2-1* mutants.

Supplementary References:

1. Zeisel, A. *et al.* Molecular Architecture of the Mouse Nervous System. *Cell* **174**, 999–1014.e22 (2018).
2. Huisman, C. *et al.* Single cell transcriptome analysis of developing arcuate nucleus neurons uncovers their key developmental regulators. *Nat. Commun.* **10**, 3696 (2019).
3. Kim, D.-W. *et al.* Multimodal Analysis of Cell Types in a Hypothalamic Node Controlling Social Behavior. *Cell* vol. 179 713–728.e17 (2019).
