# Supplementary material for: The cellular and molecular landscape of hypothalamic patterning and differentiation from embryonic to late postnatal development
Source: Nat Commun. 2020 Aug 31;11:4360. doi: 10.1038/s41467-020-18231-z (PMC7459115; doi:10.1038/s41467-020-18231-z)
Supplement: Supplementary file 2 — Description of Additional Supplementary Files [file 41467_2020_18231_MOESM2_ESM.docx]

**Description of Additional Supplementary Files**

**Supplementary Data 1. Differential gene expression between the OPC-NFO-MO in the hypothalamus.**

**Supplementary Data 2. Differential gene expression in astrocytes during hypothalamic development.**

**Supplementary Data 3. Pseudotime analysis of gene expression changes during tanycyte and ependymal cell development.**

**Supplementary Data 4. Molecular markers for subregions of the developing hypothalamus, prethalamus, and other adjacent forebrain structures, between E11.5 and E13.5 used for generation of HyDD.**

**Supplementary Data 5. scCoGAPS patterns and pattern weights for all 19,622 expressed genes on the E11-E13 dataset.**

**Supplementary Data 6. Differential gene expression between subregions of the LH, PVH, prethalamus, and VMH between E11.5 and E13.5**

**Supplementary Data 7. Differential gene expression between E12.5 control (*Foxd1^Cre/+^*) and constitutively active *Ctnnb1* overexpressing mutant (*Foxd1^Cre/+^;Ctnnb1^Ex3/+^*) samples.**

**Supplementary Data 8. Differential gene expression between E12.5 control**

**(*Nkx2-1^CreER/+^*) and *Nkx2-1*-deficient mutant (*Nkx2-1^CreER/CreER^*) samples.**

**Supplementary Data 9: Top-ranked positional markers of the developing diencephalon between E11.5 and E13.5**
